# Protospacer-Adjacent Motif Specificity during Clostridioides difficile Type I-B CRISPR-Cas Interference and Adaptation

**DOI:** 10.1128/mBio.02136-21

**Published:** 2021-08-24

**Authors:** Anna Maikova, Pierre Boudry, Anna Shiriaeva, Aleksandra Vasileva, Anaïs Boutserin, Sofia Medvedeva, Ekaterina Semenova, Konstantin Severinov, Olga Soutourina

**Affiliations:** a Center of Life Sciences, Skolkovo Institute of Science and Technology, Moscow, Russia; b Université Paris-Saclay, CEA, CNRS, Institute for Integrative Biology of the Cell (I2BC), Gif-sur-Yvette, France; c Peter the Great St. Petersburg Polytechnic University, Saint Petersburg, Russia; d Institute of Gene Biology, Centre for Precision Genome Editing and Genetic Technologies for Biomedicine, Russian Academy of Sciences, Moscow, Russia; e Waksman Institute of Microbiology, Rutgers, The State University of New Jersey, Piscataway, New Jersey, USA; f Institute of Molecular Genetics, Russian Academy of Sciences, Moscow, Russia; g Institut Universitaire de France (IUF), Paris, France; National Cancer Institute

**Keywords:** CRISPR-Cas interference, CRISPR-Cas adaptation, *Clostridium difficile*, PAM, type I-B CRISPR-Cas, enteropathogen

## Abstract

CRISPR (clustered regularly interspaced short palindromic repeats)-Cas (CRISPR-associated) systems provide prokaryotes with efficient protection against foreign nucleic acid invaders. We have recently demonstrated the defensive interference function of a CRISPR-Cas system from *Clostridioides* (*Clostridium*) *difficile*, a major human enteropathogen, and showed that it could be harnessed for efficient genome editing in this bacterium. However, molecular details are still missing on CRISPR-Cas function for adaptation and sequence requirements for both interference and new spacer acquisition in this pathogen. Despite accumulating knowledge on the individual CRISPR-Cas systems in various prokaryotes, no data are available on the adaptation process in bacterial type I-B CRISPR-Cas systems. Here, we report the first experimental evidence that the C. difficile type I-B CRISPR-Cas system acquires new spacers upon overexpression of its adaptation module. The majority of new spacers are derived from a plasmid expressing Cas proteins required for adaptation or from regions of the C. difficile genome where generation of free DNA termini is expected. Results from protospacer-adjacent motif (PAM) library experiments and plasmid conjugation efficiency assays indicate that C. difficile CRISPR-Cas requires the YCN consensus PAM for efficient interference. We revealed a functional link between the adaptation and interference machineries, since newly adapted spacers are derived from sequences associated with a CCN PAM, which fits the interference consensus. The definition of functional PAMs and establishment of relative activity levels of each of the multiple C. difficile CRISPR arrays in present study are necessary for further CRISPR-based biotechnological and medical applications involving this organism.

## INTRODUCTION

*Clostridioides* (*Clostridium*) *difficile* (1), an anaerobic, Gram-positive spore-forming bacterium, is one of the major clostridial pathogens. During the last decade, the number of severely infectious forms of C. difficile has been rising due to the emergence of hypervirulent, epidemic, and antibiotic-resistant strains (2–4). C. difficile generally causes nosocomial gut infections associated with antibiotic therapy, patient advanced age, and/or immunodeficiency (5). The incidence of community-acquired infections is on a constant rise, suggesting the importance of C. difficile reservoirs outside the hospital (6). The disturbance of gut microflora by antibiotics leads to the colonization of the intestinal tract by C. difficile cells, resulting in infection. The host immune system, the host microbiota, and its associated metabolites constitute additional factors influencing the C. difficile life cycle (7). During its infection cycle, C. difficile usually produces two toxins, TcdA and TcdB, that are major virulence factors (8). These toxins induce lysis of enterocytes and robust inflammatory reaction, which leads to diarrhea, pseudomembranous colitis, and even colon perforation and patient’s death (9–11).

Inside the gut, C. difficile forms spores, which are released into the environment where they remain infectious. C. difficile metabolically adapts to changing environments and various stresses inside the host (5) and forms biofilms (12–14). C. difficile vegetative cells also interact with phages in phage-rich gut communities (15, 16). Despite much progress in recent years, many aspects of C. difficile pathogeneses, including molecular mechanisms of the host infection, are still poorly understood.

The CRISPR (clustered regularly interspaced short palindromic repeats)-Cas (CRISPR-associated) systems provide prokaryotes with adaptive immunity against phages and other mobile genetic elements, such as plasmids and transposons (17). These defensive systems are found in almost all sequenced archaeal genomes and in approximately half of bacterial genomes (18). CRISPR-Cas systems are composed of CRISPR arrays and *cas* gene operons. CRISPR arrays consist of short direct repeat sequences (∼20 to 50 bp) (19) separated by spacers of variable sequences. Some spacers are complementary to sequences in viral genomes or in other mobile genetic elements (20). CRISPR arrays are preceded by leader regions containing promoters required for their expression and DNA sequences required for acquisition of new spacers (21–23).

The defensive function of CRISPR-Cas systems is based on two processes: interference and adaptation (24). During interference, a CRISPR array is transcribed into a precursor RNA (pre-crRNA) that is processed into small CRISPR RNAs (crRNAs), each of which consists of one spacer and flanking repeat sequences. Individual crRNAs bind certain Cas proteins forming an effector complex. This complex recognizes and cleaves foreign nucleic acids that are complementary to the spacer part of crRNA (25). During CRISPR adaptation, spacers are acquired from foreign genetic elements into CRISPR arrays, thus allowing prokaryotic cells to memorize and cope with genetic invaders (24). The Cas1 and Cas2 proteins, found in almost all investigated CRISPR-Cas systems, are essential for this process (26). The Cas4 protein present in some CRISPR-Cas systems (26) plays an important auxiliary role in adaptation (27–31).

A crucial aspect of CRISPR-based immunity is the ability to distinguish host DNA from foreign nucleic acids. Protospacer-adjacent motifs (PAMs) are short sequences located at the 3′ or 5′ end of a protospacer (i.e., the region of foreign nucleic acid corresponding to a spacer in the CRISPR array). The presence of PAM is essential for interference. Since PAMs are absent in CRISPR arrays, autoimmunity caused by self-targeting of spacers in CRISPR arrays is prevented (17). For efficient defense, spacers acquired during adaptation should be selected from protospacers with functional PAMs. Since adaptation and interference can occur independently of each other, the supply of functional interference-proficient spacers can be achieved either by counterselection of spacers acquired from sequences with nonfunctional PAMs or by coevolution of specificities of the adaptation and interference machineries toward common PAM sequences. Indeed, in cases when it has been studied, the specificity of the adaptation and interference machineries for PAM is overlapping but not fully identical (32–35).

CRISPR-Cas systems are highly diverse and classified in accordance with their *cas* operon architecture into two different classes that are further subdivided into six types and 33 subtypes (26, 35, 36). Class 1 systems, which include types I, III, and IV, are characterized by effector complexes composed of multiple Cas proteins. Class 2 systems, which include types II, V, and VI, possess a single multidomain effector Cas protein such as Cas9. C. difficile possesses an interference-proficient type I-B CRISPR-Cas system (37–40) characterized by an unusually large set of actively expressed arrays. Genome sequencing and transcriptome sequencing (RNA-seq) analysis of the reference 630 strain and hypervirulent R20291 C. difficile strain identified 12 and 9 CRISPR arrays, respectively, from which crRNAs are produced (38). Analysis of 217 C. difficile genomes revealed, on average, 8.5 CRISPR arrays per genome, some located in prophages (38–40). Another specific feature is the presence of two or even three (in 027 ribotype strains) type I-B *cas* operons in most sequenced C. difficile strains (38). We recently showed that the C. difficile 630Δ*erm* CRISPR-Cas system is capable of interference (37, 38). These studies demonstrated that individual crRNAs corresponding to different CRISPR arrays are expressed at very different levels, raising a question of differential contributions of various CRISPR arrays to defense (37, 38). We also predicted 3-nucleotide 5′ motifs CCA and CCT as PAMs and experimentally confirmed them for C. difficile 630Δ*erm* (38). Based on this knowledge, we have recently developed a new method for genome editing in C. difficile using its native CRISPR-Cas system (41). However, a global view of PAM efficiency for CRISPR interference and adaptation is still missing in C. difficile. Despite intensive studies devoted to various CRISPR-Cas systems, many questions remain unanswered on the functional features of individual CRISPR-Cas subtypes. In particular, no data are available on the adaptation process for bacterial type I-B CRISPR-Cas system. Uncovering the molecular characteristics of the CRISPR-Cas system in C. difficile is of particular importance to better understand its survival in phage-rich gut communities and for the harnessing of this efficient system for new antibacterial and genome editing applications. In the present work, we provide the first experimental evidence for spacer acquisition in C. difficile and explore the PAM requirements of C. difficile CRISPR interference and adaptation machineries.

## RESULTS

### Experimental determination of YCN as a PAM for C. difficile CRISPR-Cas system interference.

**FIG 1:**
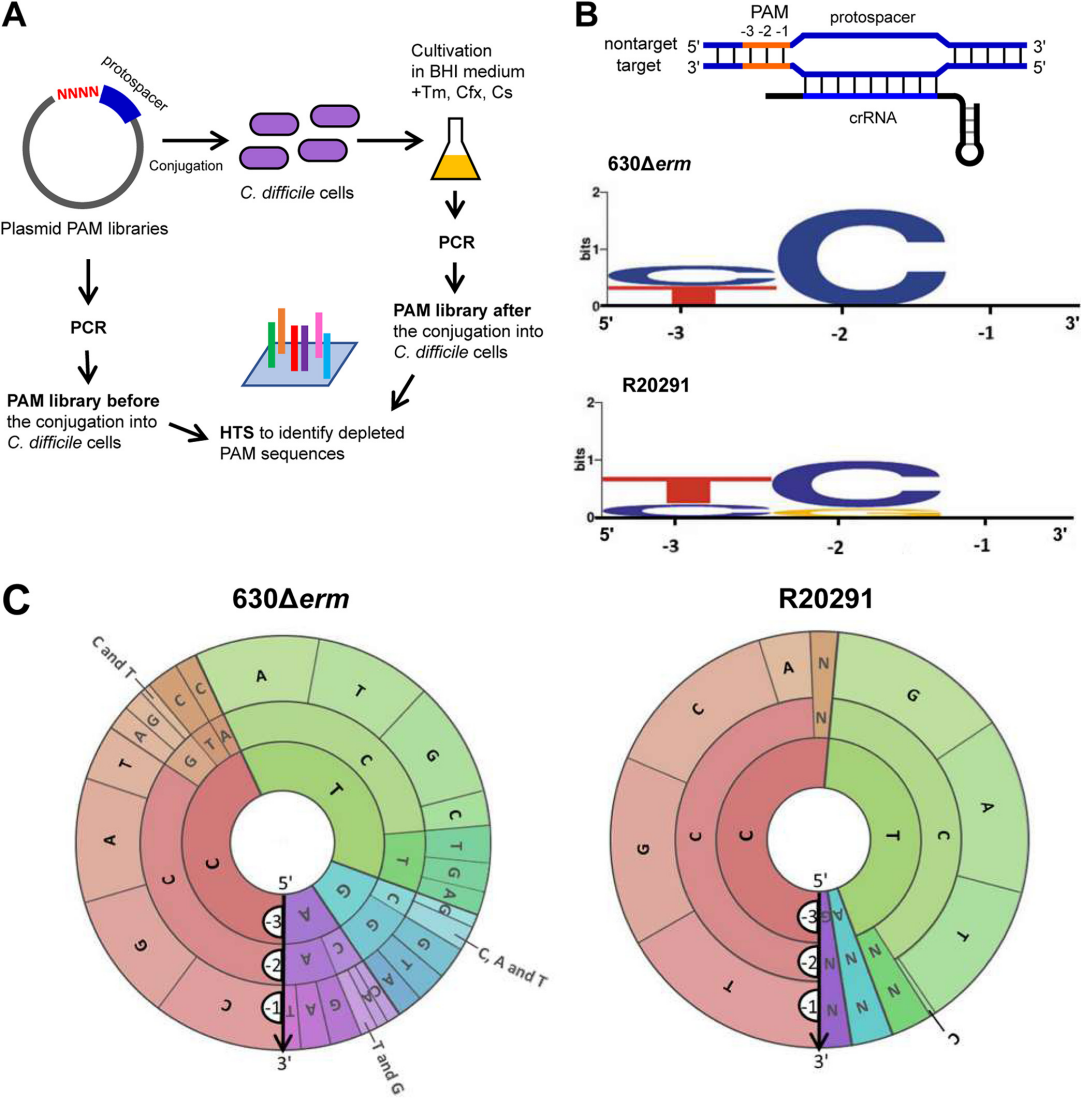
PAM sequence determination in C. difficile. (A) Experimental strategy for PAM identification using plasmid libraries in C. difficile. Tm, thiamphenicol; Cfx, cefoxitin; Cs, d-cycloserine; HTS, high-throughput sequencing. (B) WebLogos for the trinucleotide PAM consensus in C. difficile 630Δ*erm* and R20291 strains. (C) PAM wheels for C. difficile 630Δ*erm* and R20291 strains. Arrows indicate the direction (5′→3′) from the −3 nucleotide position to the −1 nucleotide position of PAMs. Red sectors correspond to the CCN PAM consensus, and green sectors correspond to the TCN PAM consensus. PAM WebLogos (B) represent the consensus of the most depleted sequences, while PAM wheels (C) visualize individual PAM sequences and their depletion scores.

To identify the PAM consensus for CRISPR interference in C. difficile 630Δ*erm* and R20291 strains, we performed conjugation depletion assays with plasmid PAM libraries (Fig. 1A). To generate potential PAM sequence variety, four randomized nucleotides were inserted upstream of a target protospacer sequence in the pRPF185Δ*gus* plasmid. Plasmids harboring the PAM library were conjugated into C. difficile, and pooled transconjugants were subjected to high-throughput sequencing. Comparison of the compositions of the input PAM library and sequences found in transconjugants allows identification of functional PAMs, since plasmids harboring functional PAM sequences would be cleared by the CRISPR interference. For construction of plasmid PAM libraries, we selected protospacers corresponding to first spacers within the CRISPR 3 (identical to CRISPR 16) array of strain 630Δ*erm* and CRISPR 13 array from strain R20291. These arrays are actively expressed, and crRNAs derived from strain 630Δ*erm* CRISPR 3/16 are capable of interference (38). After the transformation of plasmid libraries into Escherichia coli cells, ∼8,000 clones for the 630Δ*erm* plasmid library and ∼9,500 clones for the R20291 library were obtained, enough to provide >10-fold coverage of the 4-nucleotide library. Plasmids from pooled transformants were used to prepare input libraries (referred to as “PAM libraries before conjugation” in Fig. 1A). After conjugation, ∼4,000 and ∼2,000 transconjugants were obtained for 630Δ*erm* and R20291 strains, respectively. An additional subculturing step in brain heart infusion (BHI) liquid medium supplemented with antibiotics was used to eliminate remaining E. coli cells. Cells from resulting liquid cultures were collected for DNA extraction and PCR amplification to prepare output libraries (referred to as “PAM libraries after conjugation” in Fig. 1A).

For each strain, input and output libraries were subjected to high-throughput sequencing. Recovered sequences were compared using Pearson’s chi-square test to reveal PAM sequences significantly depleted after conjugation (with a *P* value of less than 10^−12^). This analysis suggested that the −4 position of PAM is not relevant for interference by the C. difficile CRISPR-Cas system (see Fig. S1 in the supplemental material). WebLogo-based visualization of the trinucleotide motifs revealed the YCN PAM consensus for both the 630Δ*erm* and R20291 strains (Fig. 1B). PAM wheels (Fig. 1С) confirmed the YCN PAM consensus.

10.1128/mBio.02136-21.1 FIG S1 WebLogos for the four-nucleotide PAM consensus motifs in C. difficile 630Δ*erm* and R20291 strains. Download FIG S1, PDF file, 0.07 MB. Copyright © 2021 Maikova et al. 2021 Maikova et al. https://creativecommons.org/licenses/by/4.0/ This content is distributed under the terms of the Creative Commons Attribution 4.0 International license.

**FIG 2:**
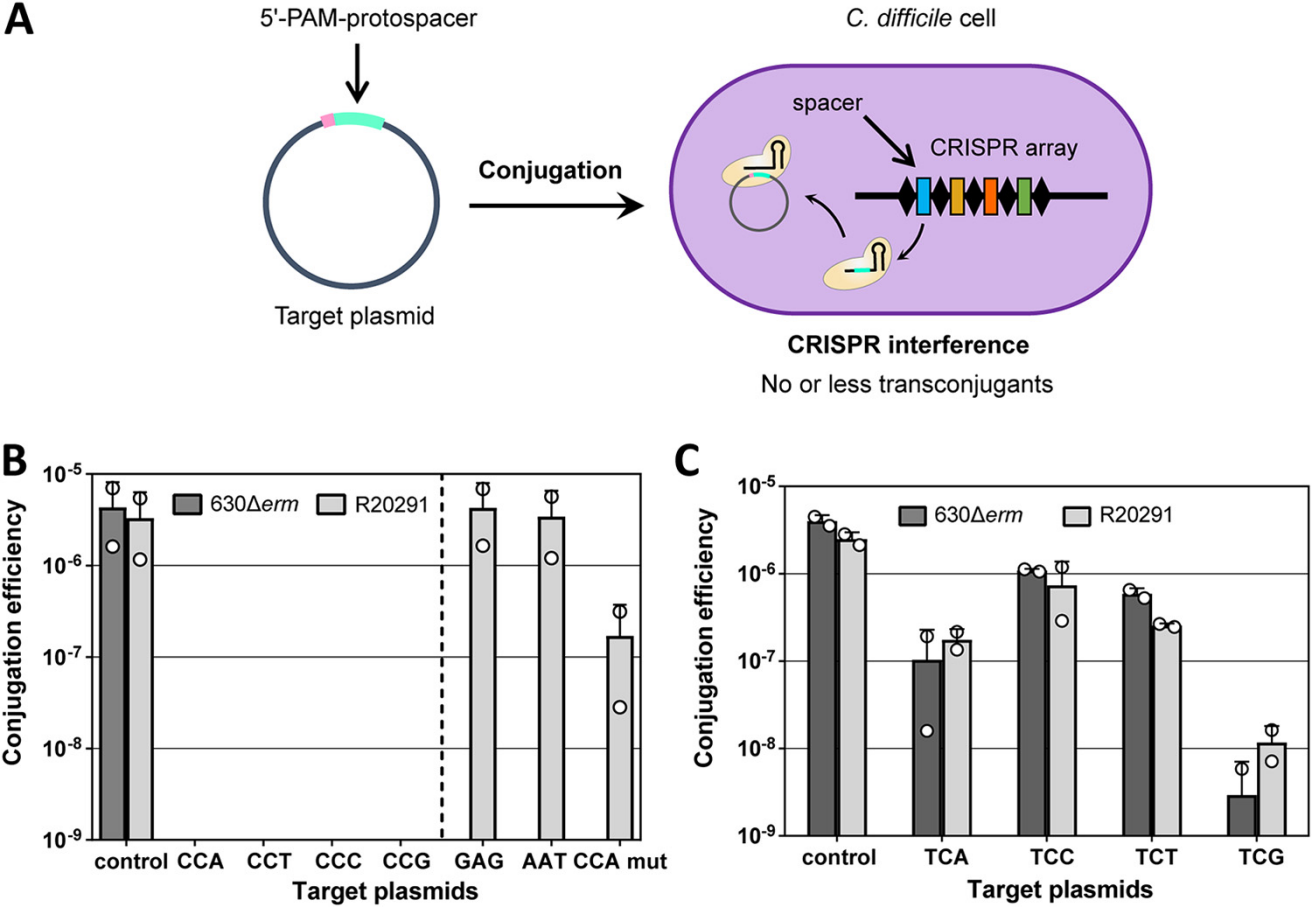
Functional PAM validation in C. difficile by plasmid interference assays. (A) Experimental strategy for plasmid interference assays. Conjugative vectors carrying 5′-PAM protospacers corresponding to a selected spacer of C. difficile CRISPR arrays were conjugated into C. difficile cells, and subsequently, the efficiency of conjugation was determined. Higher conjugation efficiency corresponds to lower interference levels. (B) Plasmid conjugation efficiencies for plasmids carrying protospacers with CCN PAMs in C. difficile 630Δ*erm* and plasmids carrying protospacers with CCN PAMs and nonfunctional PAMs (GAG, AAT) in R20291 strains. “CCA mut” depicts the plasmid carrying the CCA PAM and the protospacer, mutated at the first position. The broken line separates the results of the R20291 strain only. An empty pRPF185Δ*gus* vector was used as a conjugation control. (C) Plasmid conjugation efficiencies for plasmids carrying protospacers with TCN PAMs in C. difficile 630Δ*erm* and R20291 strains. An empty pRPF185Δ*gus* vector was used as a conjugation control. Means of results from two independent experiments are presented with individual datapoints indicated. Error bars correspond to standard deviations from two biological replicates.

The YCN PAM consensus agrees with the CCW PAM bioinformatically predicted and experimentally validated in the C. difficile 630 strain (38). To validate the functionality of PAMs determined by the PAM library depletion analysis, we constructed plasmids containing protospacers used for plasmid PAM libraries and flanked by individual YCN PAMs on the 5′ end. The set of PAM-protospacer-carrying plasmids was conjugated into C. difficile cells, and conjugation efficiency was determined. An empty pRPF185Δ*gus* vector was used as a control (Fig. 2A). Plasmids carrying CCN PAMs gave no transconjugants in both strains (Fig. 2B). This confirms that the CCN PAM is functional for interference in C. difficile. Control experiments showed that plasmids carrying a GAG trinucleotide or AAT, a sequence from the 3′ end of a CRISPR repeat, in the position of a PAM conjugated as efficiently as the pRPF185Δ*gus* vector (Fig. 2B). Mutation at the first position of the protospacer led to reduced interference against a plasmid carrying the CCA PAM in R20291, as expected (42).

In contrast to results obtained with CCN PAMs, detectable conjugation was observed with plasmids carrying TCN PAMs. Compared to that of the control, the conjugation efficiency was decreased 50- to 500-fold for TCA and TCG motifs, respectively. The TCC/T PAMs were the least effective (Fig. 2C). These results indicate that TCN sequences are generally less functional as interference PAMs than CCN.

### Varied protection efficiencies of different C. difficile 630Δ*erm* CRISPR arrays.

**FIG 3:**
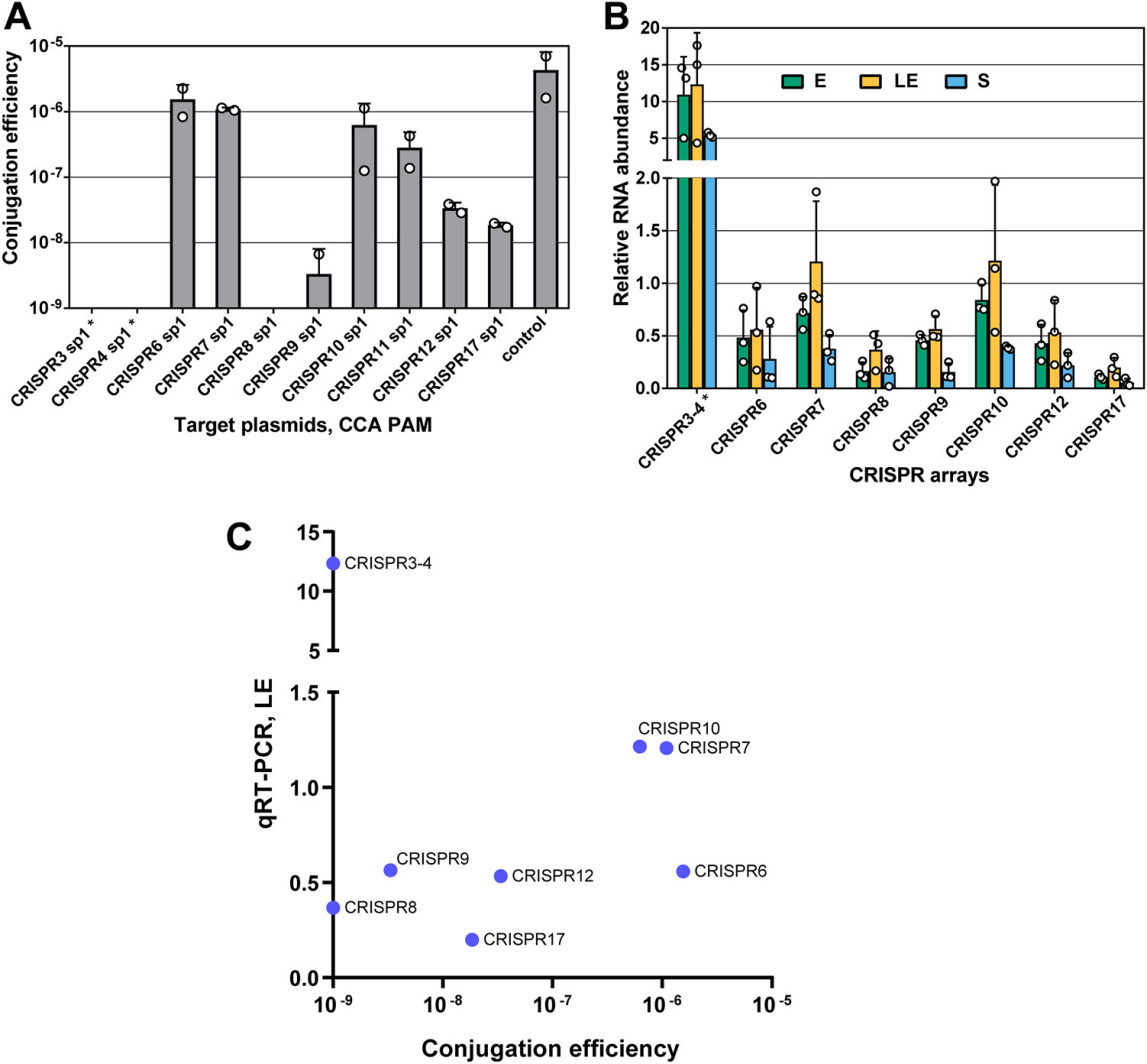
CRISPR array contribution to interference and their expression in C. difficile 630Δ*erm.* (A) Plasmid conjugation efficiencies in C. difficile strain 630Δ*erm*. Plasmids carrying different protospacers corresponding to each C. difficile 630Δ*erm* spacer and flanked by CCA PAM were used. An empty pRPF185Δ*gus* vector was used as a conjugation control. Data are the means from two biological replicates, with individual datapoints indicated. (B) qRT-PCR analysis of CRISPR array expression in C. difficile 630Δ*erm* in exponential (E), late exponential (LE), and stationary (S) phases of culture growth. Forward primers were annealed to the leader sequence of each array, and reverse primers were annealed to the first spacer of each array. CRISPR 3 and CRISPR 4 are cotranscribed and presented as CRISPR 3-4. Values represent means ± standard deviations (*N *= 3). (C) Plasmid conjugation efficiencies versus expression levels defined by qRT-PCR in the late exponential (LE) phase of the culture growth. Plasmids carrying protospacers corresponding to spacers of CRISPR 3/16, CRISPR 4/15, and CRISPR 8 gave no transconjugants; therefore, their conjugation efficiencies are less than or equal to 10^−9^. *, sequences of CRISPR 3-4 and CRISPR 16-15 arrays of C. difficile strain 630Δ*erm* are identical; therefore, CRISPR 16 and 15 are not presented.

A specific feature of the C. difficile CRISPR-Cas system is the presence of multiple actively expressed CRISPR arrays. The functionality of all 12 CRISPR arrays in the C. difficile 630Δ*erm* strain was investigated using plasmids containing protospacers corresponding to selected spacers from each of C. difficile 630Δ*erm* CRISPR arrays and flanked by a functional CCA PAM on the 5′ end. The cotranscribed CRISPR 3-4 and CRISPR 16-15 arrays located in homologous prophages phiCD630-1 and phiCD630-2 are identical to each other and are thus indistinguishable by this experimental strategy (37). Plasmids carrying protospacers corresponding to spacers of CRISPR 3/16, CRISPR 4/15, and CRISPR 8 gave no transconjugants (Fig. 3A and Fig. S2A). Strongly reduced conjugation efficiency was observed with plasmids carrying protospacers corresponding to spacers from CRISPR 9, CRISPR 12, and CRISPR 17 arrays. Surprisingly, the conjugation efficiency for plasmids carrying protospacers corresponding to spacers of CRISPR 6 and CRISPR 7 was close to that of the control vector (Fig. 3A). Thus, not all C. difficile 630Δ*erm* CRISPR arrays are equally functional for interference.

10.1128/mBio.02136-21.2 FIG S2 (A) Plasmid conjugation efficiencies in C. difficile strain 630Δ*erm*. Plasmids carrying protospacers corresponding to C. difficile 630Δ*erm* spacers 1, 3, and 6 from CRISPR 3 array and spacers 1, 3, and 6 from CRISPR 12 flanked by CCA PAM were used. An empty pRPF185Δ*gus* vector was used as a conjugation control. Data are the means from two biological replicates, with individual datapoints indicated. (B) Expression levels of 630Δ*erm* strain CRISPR 3-4 and CRISPR 12 arrays detected by RNA-seq in our previous work (38). Red arrows marked with “+1” designate the transcriptional start site of the arrays and correspond to the leader sequences; black diamonds point to the beginnings of the arrays. Spacers are marked with red-colored blocks. Download FIG S2, PDF file, 0.09 MB. Copyright © 2021 Maikova et al. 2021 Maikova et al. https://creativecommons.org/licenses/by/4.0/ This content is distributed under the terms of the Creative Commons Attribution 4.0 International license.

We wondered whether these differences in interference could be correlated with the relative expression levels of corresponding crRNAs. Those most active for interference CRISPR 3/16, CRISPR 4/15, CRISPR 8, and CRISPR 9 arrays (Fig. 3A) are also the most highly expressed in the 630Δ*erm* strain (37, 38). Within the same CRISPR array, the most-abundant sequence reads mapped to the leader-proximal regions, i.e., closer to promoters from which arrays are transcribed (37, 38). The decreasing gradient in the amounts of crRNAs observed for the first, third, and sixth spacer from the CRISPR 12 array is consistent with the interference levels provided by these crRNAs (Fig. S2), supporting a notion that the abundance of crRNAs produced from different arrays or from within the same array affects the level of interference.

We next compared the interference levels provided against protospacer plasmids (Fig. 3A) and the expression levels of each C. difficile 630Δ*erm* CRISPR array as measured by reverse transcription-quantitative PCR (qRT-PCR) (Fig. 3B). As expected, the arrays that were most active for interference (CRISPR 3/16 and CRISPR 4/15) were the most highly expressed (Fig. 3A to C). However, CRISPR 6 and CRISPR 7 arrays, which provided low protection against plasmid conjugation, were expressed at levels similar to those of other active arrays, i.e., CRISPR 8 and CRISPR 9. These results suggest that besides CRISPR array expression levels, additional mechanisms, potentially related to sequences of individual crRNAs, control the activity of CRISPR arrays in C. difficile.

### Active spacer acquisition by the C. difficile CRISPR-Cas I-B system.

**FIG 4:**
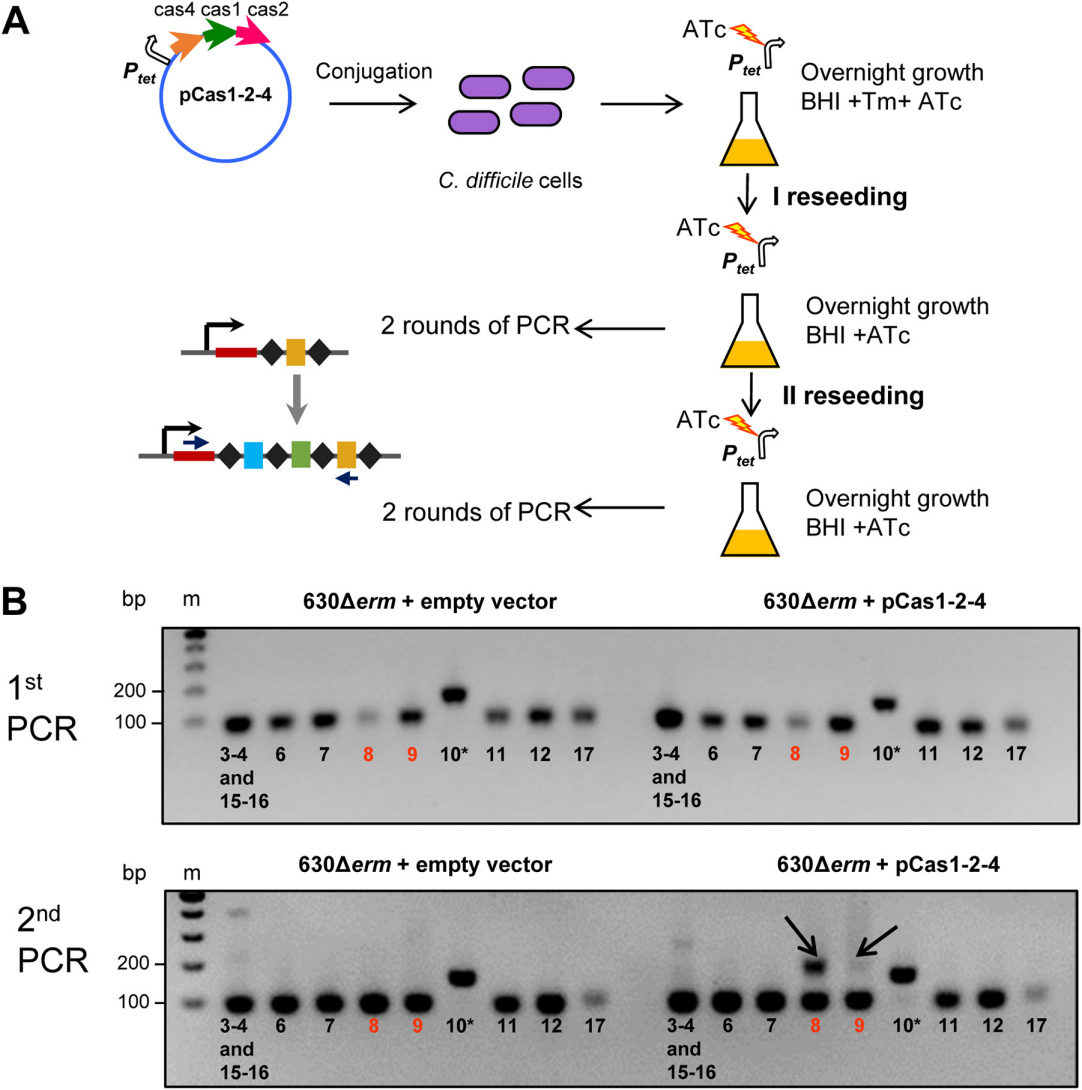
Adaptation experiment in C. difficile 630Δ*erm.* (A) Experimental workflow of naive adaptation assays in C. difficile 630Δ*erm* using pCas1-2-4 overexpressing Cas1, Cas2, and Cas4 proteins. Two reseeding steps after cultivation of transconjugants in BHI plus Tm plus ATc medium and following two rounds of PCR amplification (“1st PCR” and “2nd PCR”) were performed for the detection of extended CRISPR arrays. PCR amplification was performed using pairs of primers for each CRISPR array of C. difficile 630Δ*erm*. Forward primers annealed to leader regions of arrays, and reverse primers annealed to the first or the second spacer (CRISPR 10 array) of a native array. Tm, thiamphenicol; ATc, anhydrotetracycline. (B) PCR analysis of naive adaptation in C. difficile 630Δ*erm*. First and second PCR results after the II reseeding step are presented. Numbers bellow PCR bands denote C. difficile 630Δ*erm* CRISPR arrays (CRISPR 3-4, CRISPR 6, etc.); 89-bp PCR bands correspond to native arrays (*155 bp for CRISPR 10 array); 155-bp PCR bands correspond to one acquired spacer (*221 bp for CRISPR 10 array). Sequences of CRISPR 3-4 and CRISPR 16-15 arrays are identical; therefore, they are presented at the same lanes. PCR bands corresponding to new spacer acquisition are marked with arrows. Lane m, molecular mass markers.

We next investigated the ability of C. difficile CRISPR-Cas to take up new spacers. All attempts to detect expansion of CRISPR arrays in C. difficile cells grown under laboratory conditions were unsuccessful. We hypothesized that endogenous expression levels of Cas proteins could be insufficient for adaptation. Therefore, we constructed two plasmids carrying *cas* genes from the adaptation module under the control of an inducible P*_tet_* promoter (see Table S1). The first plasmid (pCas1-2) carried the *cas1* and *cas2* genes encoding universal CRISPR adaptation proteins, whereas the second plasmid (pCas1-2-4) carried *cas1*, *cas2*, and *cas4* genes, thus encoding the complete adaptation module. We next tested the ability of C. difficile 630Δ*erm* transformed with these plasmids to take up new spacers. Cells carrying an empty vector were used as a control. Transconjugants were cultivated in a medium supplemented with an inducer of *cas* gene expression. No growth of the strain carrying pCas1-2 was observed, presumably due to a toxic effect of overexpression of Cas1 and Cas2. In contrast, cells carrying pCas1-2-4 grew well after induction. We tested each of the 12 C. difficile 630Δ*erm* CRISPR arrays for signs of spacer acquisition by using PCR with one primer annealing to the leader and another annealing to a preexisting spacer within the array (Fig. 4A). PCR products corresponding to expanded arrays with one additional spacer were observed only for CRISPR 8 and CRISPR 9 in cells overexpressing the adaptation module but not in control cells (Fig. 4B).

10.1128/mBio.02136-21.7 TABLE S1 Bacterial strains and plasmids used in this study. Download Table S1, PDF file, 0.1 MB. Copyright © 2021 Maikova et al. 2021 Maikova et al. https://creativecommons.org/licenses/by/4.0/ This content is distributed under the terms of the Creative Commons Attribution 4.0 International license.

### High-throughput analysis of newly acquired spacers.

**FIG 5:**
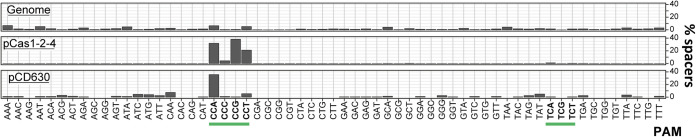
Distribution of PAM sequences corresponding to uniquely acquired spacers in C. difficile 630Δ*erm*. Functional PAM sequences are indicated in bold and underlined in green.

**FIG 6:**
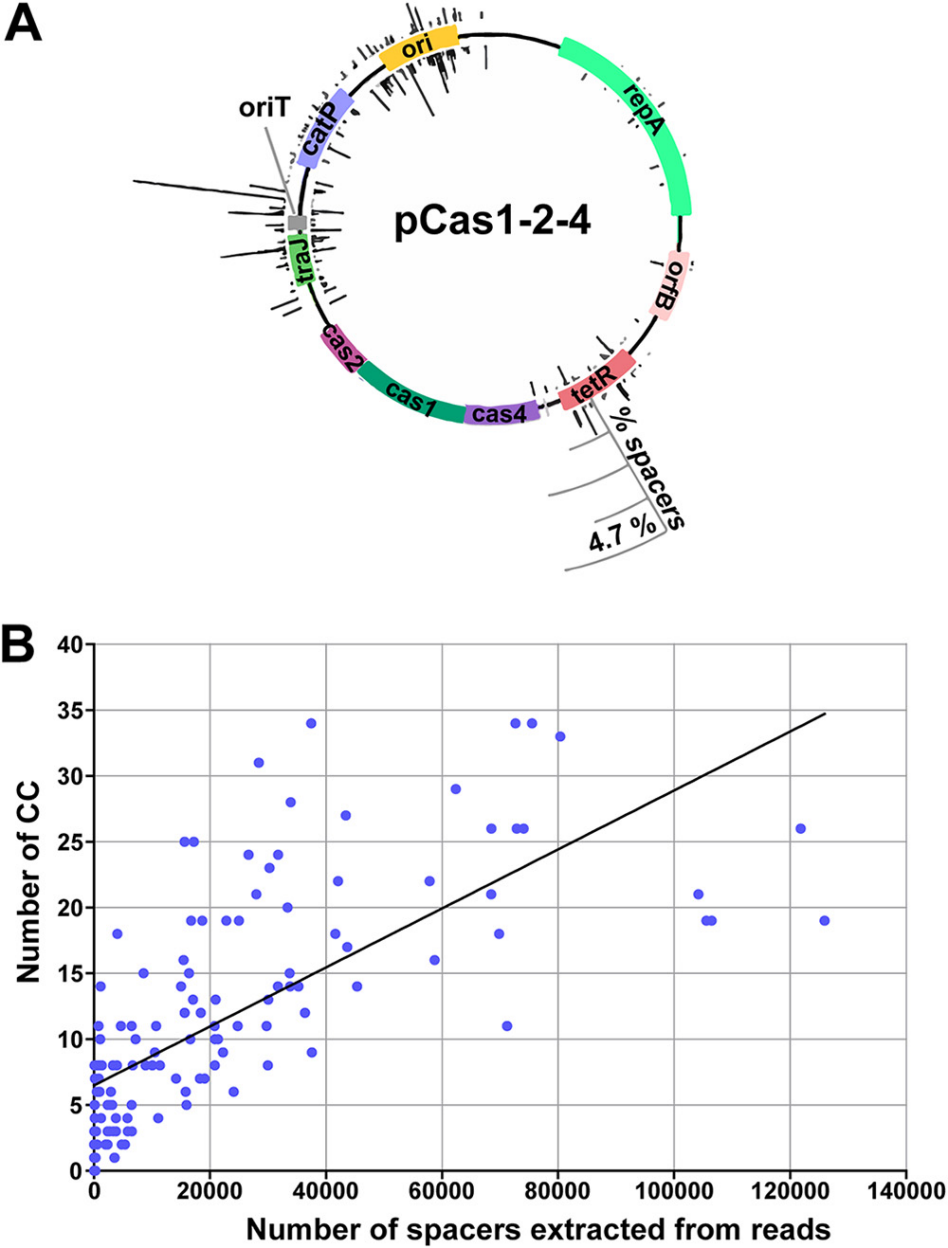
Analysis of newly acquired unique spacers mapped to the plasmid pCas1-2-4 in C. difficile 630Δ*erm*. (A) Distribution of spacers aligned to the pCas1-2-4. The heights of the black bars indicate the percentages of reads containing the corresponding spacer aligned to certain positions on the plasmid; 100% corresponds to all spacer reads mapped to either the genome, pCas1-2-4, or pCD630. Bars inside and outside the plasmid circles indicate spacers derived from different strands of DNA. (B) Numbers of CC nucleotides and the numbers of spacers extracted from CRISPR array reads and mapped to 300-nt bins on the pCas1-2-4 plasmid.

DNA products corresponding to expanded CRISPR arrays were subjected to high-throughput sequencing to identify the sources of new spacers. Overall, 299,674 unique newly acquired spacers were extracted and mapped to the three spacer sources (the chromosome, the pCas1-2-4 plasmid, and the endogenous pCD630 plasmid present in the C. difficile 630Δ*erm* strain [43]) (Table S4). We analyzed the lengths of acquired spacers, the distribution of corresponding protospacers along the chromosome and plasmids, and associated PAM sequences. Almost all newly acquired spacers, irrespective of their source, were 34 to 40 bp in length (36 to 37 bp average). This size distribution matches well with that of spacers preexisting in C. difficile 630Δ*erm* arrays (see Fig. S3). The largest numbers of spacers (98% of uniquely mapped spacers) were derived from the pCas1-2-4 plasmid (Table S4; Fig. S4). The majority of pCas1-2-4-derived spacers (96%) originated from sequences associated with the CCN PAM (Fig. 5). Spacers mapped to pCas1-2-4 nonuniformly (Fig. 6A). The most frequent spacers were acquired from the *traJ* gene encoding a regulator of conjugative gene expression, the *oriT* region for plasmid transfer, and the *ori* region for plasmid replication (Fig. 6A). The bias in protospacer distribution along the pCas1-2-4 plasmid could be explained, at least partially, by nonuniform density of the CC dinucleotide (Fig. 6B), since the number of protospacers selected from different pCas1-2-4 regions correlated with the number of CC dinucleotides in these regions (*R* = 0.72).

10.1128/mBio.02136-21.3 FIG S3 Distribution of spacer lengths. (A) Lengths of spacers acquired by CRISPR 8 and CRISPR 9 arrays during the adaptation. (B) Lengths of native spacers in C. difficile 630Δ*erm* CRISPR arrays. Download FIG S3, PDF file, 1.1 MB. Copyright © 2021 Maikova et al. 2021 Maikova et al. https://creativecommons.org/licenses/by/4.0/ This content is distributed under the terms of the Creative Commons Attribution 4.0 International license.

10.1128/mBio.02136-21.4 FIG S4 Distribution of spacers, aligned to the chromosome (A), pCas1-2-4 plasmid (B), and pCD630 plasmid (C). The heights of black bars indicate the percentages of spacers aligned to certain positions on DNA molecules. Bars above and below chromosome line (A) and inside and outside plasmid circles (B and C) designate spacers derived from different strands of DNA. Red bars indicate nonuniquely aligned spacers. Most of the spacers targeting both genomic or pCas1-2-4 DNA molecules (“nonunique spacers”) were derived from *cas1*, *cas2*, or *cas4* genes present in the chromosome and pCas1-2-4. Considering the general paucity of chromosome-derived spacers, most *cas1*, *cas2*, and *cas4* matching spacers must be acquired from the plasmid. Download FIG S4, PDF file, 0.5 MB. Copyright © 2021 Maikova et al. 2021 Maikova et al. https://creativecommons.org/licenses/by/4.0/ This content is distributed under the terms of the Creative Commons Attribution 4.0 International license.

10.1128/mBio.02136-21.10 TABLE S4 Number of spacers acquired into CRISPR 8 and CRISPR 9 arrays. The table shows the total number of reads, the number of aligned spacers, and the number of spacers mapped to different DNA molecules (chromosome, pCas1-2-4, and pCD630). Spacers that have multiple alignments but on different molecules (nonunique alignment) are shown separately. Percentages of spacers used in further analysis are shown in red. Download Table S4, PDF file, 0.06 MB. Copyright © 2021 Maikova et al. 2021 Maikova et al. https://creativecommons.org/licenses/by/4.0/ This content is distributed under the terms of the Creative Commons Attribution 4.0 International license.

Among endogenous DNA sources, 0.07% of uniquely mapped spacers originated from the 7,881-bp endogenous pCD630 plasmid (Table S4; Fig. S4C). The CCA motif was found at the highest frequency upstream of pCD630-derived protospacers, suggesting that, similarly to pCas1-2-4, interference-proficient spacers can be selected from pCD630, preserved in the cell population, and presumably lead to plasmid loss (Fig. 5). The adaptation enrichment regions for pCD630 were localized at the region near the *p70* gene encoding a putative enzyme of the helicase family and close to the region of the *p80* gene encoding a hypothetical protein.

**FIG 7:**
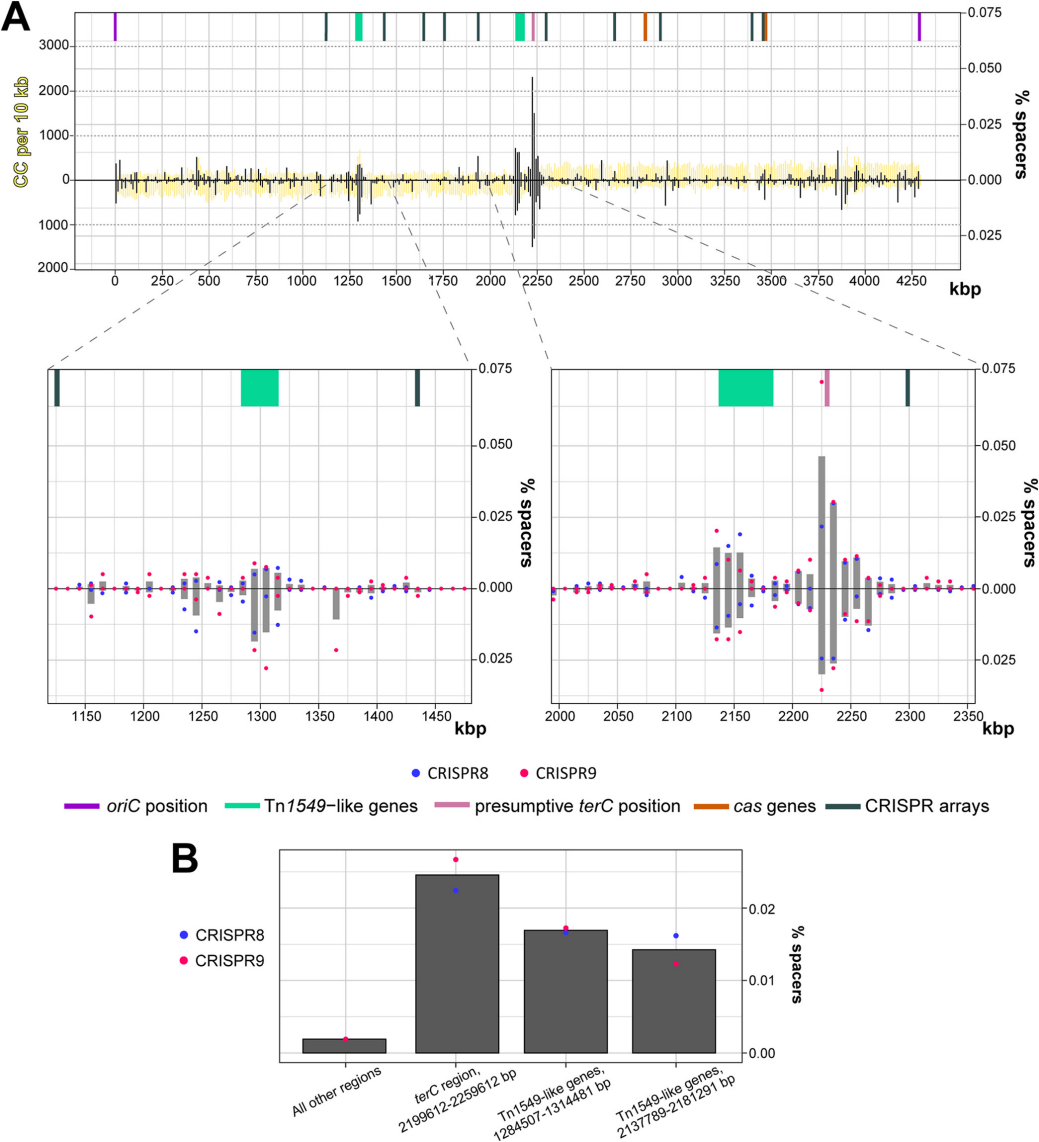
Analysis of newly acquired unique spacers mapped to the C. difficile 630Δ*erm* chromosome. (A) Distribution of spacers derived from the chromosome. The most-spacer-enriched regions are scaled up. The heights of black bars indicate the percentages of reads containing spacers mapped to 10-kbp nonoverlapping genomic regions aligned to certain positions on the DNA molecule; 100% corresponds to all spacer reads mapped to either the genome, pCas1-2-4, or pCD630. Bars localized above and below the chromosome line designate spacers derived from different strands of DNA. The heights of yellow bars show the frequencies of CC dinucleotides on the chromosome. Blue and red dots designate the percentages of spacers acquired to CRISPR 8 and CRISPR 9 arrays, respectively. The position of *oriC* is as defined in reference 70. (B) Spacer acquisition efficiencies of chromosomal regions. Blue and red dots designate the percentages of spacers acquired to CRISPR 8 and CRISPR 9 arrays, respectively.

Only a small fraction of uniquely mapped spacers (1.69%) was acquired from the 4,290,252-bp bacterial genome (Table S4). In contrast to pCas1-2-4 and pCD630 protospacers, no overrepresented trinucleotide motif was found upstream of protospacers derived from the chromosome (less than 10% were associated with the CCN or TCN PAMs). The differences in PAM specificity between genome- and plasmid-derived protospacers must be caused by lethality of spacers derived from genomic protospacers with interference-proficient PAMs (Fig. 5). In other words, the genome-derived spacers that we observed must be aberrant and correspond to rare events of selection of nonfunctional spacers. Comparison of the distributions along the genome of functional and nonfunctional protospacers (see Fig. S5) suggests that selection of nonfunctional spacers occurs predominantly from regions that could serve as a source of functional spacers as well, but these functional PAM-associated spacers are depleted because of autoimmunity. Chromosomal regions that were preferentially used as donors of spacers for both CRISPR 8 and CRISPR 9 arrays included the *terC* replication termination site and loci carrying the Tn*1549*-like transposon genes (Fig. 7). This result is consistent with preferential spacer acquisition from regions prone to the generation of free DNA termini, as was also reported for other CRISPR-Cas systems (44–46).

10.1128/mBio.02136-21.5 FIG S5 Distributions of protospacers with functional and nonfunctional PAMs along the genome. The heights of black bars indicate the percentages of reads containing spacers mapped to 10-kbp nonoverlapping genomic regions aligned to certain positions on the DNA molecule; 100% corresponds to all spacer reads mapped to either the genome, pCas1-2-4, or pCD630. Bars above and below chromosome line designate spacers derived from different strands of DNA. Download FIG S5, PDF file, 0.3 MB. Copyright © 2021 Maikova et al. 2021 Maikova et al. https://creativecommons.org/licenses/by/4.0/ This content is distributed under the terms of the Creative Commons Attribution 4.0 International license.

## DISCUSSION

CRISPR-Cas systems provide prokaryotes with adaptive immunity by recognizing and specifically eliminating invaders, such as viruses and plasmids. The CRISPR-Cas systems are highly diverse, and detailed studies of individual CRISPR-Cas subtypes continue to reveal features important for understanding of various aspects of microbial physiology as well as for the potential biotechnological and medical applications. In this study, we provide the first experimental evidence for type I-B CRISPR-Cas system adaptation in an important human pathogen, C. difficile, and reveal a functional link between the adaptation and interference machineries by demonstrating preferential selection of newly acquired spacers from protospacers associated with interference-proficient PAMs.

PAM library experiments allowed us to determine a general PAM consensus sequence (YCN) for the C. difficile CRISPR-Cas system in both the laboratory 630Δ*erm* strain and the hypervirulent R20291 strain. These results are in accordance with CCW PAM identification data obtained *in silico* by the alignment of existing spacers and matching protospacers and with experimental data on plasmid conjugation efficiency in C. difficile 630Δ*erm* (38). Our global approach allowed us to add TCN sequences to the list of functional C. difficile PAMs, albeit such sequences support lower levels of interference than CCN sequences. Multiple PAM sequences are recognized by type I-B systems from other sources (47). For example, in the I-B CRISPR-Cas of Haloferax volcanii, seven trinucleotide 5′-PAM motifs (TAA, TAG, TAT, TAC, TTC, ACT, and CAC) were shown to support efficient interference (48–50). The recognition of multiple PAMs is suggested to be an advantageous strategy to cope with the diversity and mutational evasion of viral invaders.

The PAM requirements and proteins involved in motif recognition differ during interference and adaptation (47). In H. volcanii type I-B CRISPR-Cas, only the TAC PAM was associated with spacer acquisition, suggesting a PAM recognition mechanism that is stricter for adaptation than for interference (45). The PAM requirements for spacer acquisition remained unexplored in bacterial I-B CRISPR-Cas systems.

In general, the spacer acquisition process in various CRISPR-Cas systems has been extensively studied, including recent reports on naive, self-targeting-induced, and primed adaptation for archaeal type I-B CRISPR-Cas (45, 46, 51–53). However, no data on the bacterial type I-B CRISPR-Cas spacer acquisition have been reported so far. In the present study, no spacer acquisition in C. difficile was detected under the native conditions of Cas adaptation protein expression. Overexpression of Cas1, Cas2, and Cas4 proteins from a plasmid was necessary to observe the CRISPR array expansion. Cas1 and Cas2 are universal adaptation proteins. Cas4 is a part of the adaptation complex of the CRISPR-Cas systems, in which it is present (30). This protein participates in the selection and processing of prespacers, defines the correct PAM, and provides the correct orientation for new spacers during their integration into the CRISPR array (27–31). Recent studies in Pyrococcus furiosus and H. volcanii showed that the overexpression of Cas1, Cas2, and Cas4 elevates basal adaptation levels (45, 46). However, overexpression of the adaptation proteins resulted in PAM-independent acquisition in H. volcanii (45), which is clearly distinct from our observations in C. difficile.

The majority of new spacers in C. difficile 630Δ*erm* are acquired from protospacers containing the CCN PAMs, which are most efficient for interference. In the H. volcanii I-B CRISPR-Cas, only TAC PAM was associated with acquired spacers (45), suggesting that in this organism, like in C. difficile, PAM recognition requirements during spacer adaptation are stricter than those during interference. A CCN motif critical for both invader targeting and spacer DNA uptake was found upstream of protospacers from which newly acquired spacers were derived in the hyperthermophilic archaeon Pyrococcus furiosus I-B CRISPR-Cas (46).

Systematic monitoring of plasmid conjugation efficiency targeted by crRNAs from each of the 12 C. difficile 630Δ*erm* CRISPR arrays demonstrated that almost all CRISPR arrays are active for interference. However, the defense levels differed for individual CRISPR arrays, with most protective arrays generally being the most highly expressed (38). New spacer acquisition was observed only for arrays CRISPR 8 and CRISPR 9, which are among the most active for interference. The leader sequence was shown to be important for adaptation (21–23). The alignment of leader sequences of C. difficile 630Δ*erm* CRISPR arrays revealed two conserved inverted repeat motifs (see Fig. S6 in the supplemental material). These motifs were present upstream of every array, and we did not find any distinguishing features in CRISPR 8 and CRISPR 9 array leaders that would explain their adaptation proficiency (Fig. S6). Whether specific conditions could induce the expression of other CRISPR arrays and their activity for interference and spacer acquisition remain to be explored.

10.1128/mBio.02136-21.6 FIG S6 Alignment of CRISPR leader regions from C. difficile 630Δ*erm*. CRISPR 8 and CRISPR 9 arrays are highlighted with a dark-blue frame. Violet and light blue arrows designate inverted repeats that could form additional hairpins. Download FIG S6, PDF file, 0.6 MB. Copyright © 2021 Maikova et al. 2021 Maikova et al. https://creativecommons.org/licenses/by/4.0/ This content is distributed under the terms of the Creative Commons Attribution 4.0 International license.

The origins for spacer DNA uptake differ greatly in various CRISPR-Cas systems. For the type I-E system in E. coli, the acquisition of new spacers has been observed mainly from plasmids rather than from chromosomal DNA (44). In contrast, in P. furiosus, the majority of spacers were acquired from the chromosomal DNA and not from the introduced plasmids (46). In the present study, in C. difficile, the majority of new spacers were derived from the pCas1-2-4 plasmid used to express the adaptation module genes. The remaining spacers were acquired from the chromosome and the pCD630 plasmid native to C. difficile 630Δ*erm*. It is interesting that in the case of E. coli, plasmids expressing *cas1* and *cas2* tend to be preferred substrates for new spacer selection irrespective of plasmid replication origin/copy number, suggesting that some in-*cis* mechanisms may be responsible for the observed bias.

In contrast to spacers derived from plasmids, functional PAMs were not overrepresented in spacers derived from the C. difficile chromosome. This and the low number of the chromosome-derived spacers compared to the number of spacers derived from plasmids suggest that cells in which most of the spacers were acquired from chromosomal protospacers with functional PAMs were lost due to autoimmunity. Both the occurrence of PAMs and the generation of free DNA termini have emerged as features important for adaptation. For the E. coli type I-E CRISPR-Cas system, spacer acquisition was shown to preferentially occur around the chromosomal replication terminus and active CRISPR arrays where the formation of double-stranded breaks is expected (44). Similarly, in P. furiosus and H. volcanii, “hot spots” of spacer acquisition were located at sites with transposon or recombination activity, at active CRISPR loci, or in highly transcribed regions (45, 46). The situation appears to be similar in C. difficile, at least based on the distribution of the subset of genome-derived spacers that do not cause autoimmunity.

Our observations of low adaptation efficiency in *C. difficile* under normal conditions raised the question about the mechanisms that could exist to control new-DNA uptake capacities of its CRISPR-Cas system. We can hypothesize that new spacer acquisition should be limited to avoid deleterious self-targeting. This is in line with the suggestion that the adaptation machinery should be repressed under standard conditions to prevent accidental or random spacer acquisition from the genome (52). In C. difficile, we have recently shown that the *cas* operons belong to the general stress response sigma B regulon, suggesting that their expression could be induced under stressful conditions (54, 55). It is intriguing that overexpression of only Cas1 and Cas2 was highly toxic to C. difficile. A possible reason could be an indiscriminate high level of adaptation of self-targeting spacers. Clearly, this observation warrants further investigation, which, however, is complicated by the inability to obtain the needed transconjugants.

## MATERIALS AND METHODS

### Bacterial strains and growth conditions.

Bacterial strains used in this study are listed in Table S1 in the supplemental material. C. difficile strains were grown in brain heart infusion (BHI) (Difco) medium at 37°C under anaerobic conditions (5% H_2_, 5% CO_2_, and 90% N_2_), within an anaerobic chamber (Jacomex). When needed, thiamphenicol (Tm) at the final concentration of 15 μg/ml was added to C. difficile cultures. Cefoxitin (Cfx) and d-cycloserine (Cs) were used for counterselection of E. coli donor cells during conjugation into C. difficile. E. coli strains were grown in LB medium (56), supplemented with ampicillin (Amp) (100 μg/ml) and chloramphenicol (Cm) (15 μg/ml) when it was necessary. The nonantibiotic analogue anhydrotetracycline (ATc) at a concentration of 250 ng/ml was used for induction of the inducible P*_tet_* promoter of pRPF185 vector derivatives in C. difficile (57).

### Construction of plasmids and conjugation into C. difficile.

Plasmids and oligonucleotides used in this work are presented in Table S1 and Table S2, respectively. To construct plasmid PAM libraries, we used the pRPF185Δ*gus* vector. Single-stranded synthetic oligonucleotides containing four random nucleotides on the 5′ end, a selected protospacer sequence corresponding to the first spacer of CRISPR 3 (identical to CRISPR 16) or CRISPR 13 arrays for C. difficile 630 and R20291 strains, respectively, and regions overlapping the pRPF185Δ*gus* vector (37) were synthesized. Subsequently, these single-stranded synthetic oligonucleotides were amplified by PCR using short complementary primers to generate the double-stranded fragments (Table S2). To generate the PAM libraries, the double-stranded fragments were cloned into SacI and BamHI sites of pRPF185Δ*gus* using a Gibson assembly reaction (New England BioLabs) (58).

10.1128/mBio.02136-21.8 TABLE S2 Oligonucleotides used in this study. Download Table S2, PDF file, 0.2 MB. Copyright © 2021 Maikova et al. 2021 Maikova et al. https://creativecommons.org/licenses/by/4.0/ This content is distributed under the terms of the Creative Commons Attribution 4.0 International license.

For CRISPR-Cas interference assays, the synthetic complementary (5′→3′ and 3′→5′) single-stranded oligonucleotides containing SacI and BamHI restriction sites and different PAM and protospacer sequences were used to construct conjugative plasmid vectors carrying PAM-protospacer sequences. The single-stranded oligonucleotides were annealed to each other, and the resulting double-stranded fragments were ligated into SacI and BamHI sites of the pRPF185Δ*gus* vector.

To create plasmids overexpressing Cas proteins for naive adaptation assays, C. difficile 630Δ*erm cas1-cas2* and *cas4-cas1-cas2* gene regions, including ribosome-binding sites (−21 to +1252 relative to translational start site of *cas2* gene and −37 to +1773 relative to translational start site of *cas4* gene, respectively) were amplified by PCR and introduced into SacI and BamHI sites of pRPF185Δ*gus* under the control of the ATc-inducible P*_tet_* promoter, resulting in pCas1-2 and pCas1-2-4 plasmids (Table S1).

DNA sequencing was conducted to confirm the plasmid construction. All resulting plasmids were transformed into E. coli strain HB101 (RP4). E. coli transformants were subsequently mated with C. difficile cells on BHI agar plates for 24 h at 37°C. C. difficile transconjugants were selected on BHI agar containing Tm (15 μg/ml), d-cycloserine (Cs) (25 μg/ml), and cefoxitin (Cfx) (8 μg/ml).

### Conjugation with PAM libraries and high-throughput sequencing.

Plasmid PAM libraries were transformed into E. coli NEB10 beta cells (New England BioLabs). A sufficient number of Cm-resistant colonies (8,000 to 9,000) was selected and used for plasmid DNA extraction. This DNA served as a template for PCR with primers carrying Illumina adaptors, giving the control DNA sample for input libraries (named “PAM libraries before the conjugation”).

For output library preparation, the plasmid PAM libraries were transformed into E. coli HB101 RP4 cells for further conjugation into C. difficile cells (approximately 4.9 × 10^10^ plasmid copies for the 630Δ*erm* library and 2.8·10^10^ plasmid copies for the R20291 library). A sufficient number of Tm-resistant transconjugants (up to 4,000) was selected. All the transconjugants were then transferred to liquid BHI medium supplemented with antibiotics to eliminate remaining E. coli cells. Tm was used to maintain plasmids within C. difficile cells, while Cfx and Cs were used to counterselect E. coli cells sensitive to these antibiotics. Cells from the resulting liquid cultures were collected and used for the preparation of InstaGene (Bio-Rad) extracts that served as a template for PCR amplification with primers carrying Illumina adaptors, giving the DNA sample for sequencing named “PAM libraries after the conjugation.”

The DNA samples “PAM libraries before the conjugation” and “PAM libraries after the conjugation” were sequenced using an Illumina NextSeq 500 system with 2-million-read coverage. Sequence reads were aligned with reference sequences using BWA software (59). All unmapped reads were discarded from the analysis. Randomized PAM regions in selected reads were extracted using a custom-written Python script (version 3.4).

The numbers of each PAM counts were compared for two libraries (Table S3A and B). Significantly depleted PAM sequences were determined using Pearson’s chi-square test. *P* values adjusted using standard multiple testing corrections kept all possible PAM variants as depleted. Therefore, we used a *P* value of 10^−12^ to filter the highly depleted PAMs. The depleted sequences were assembled in a special data set, where the number of counts for each PAM was normalized to that of the lowest depleted PAM. The consensus of resulting sequence subsets was then visualized using the WebLogo tool (60). For the additional PAM sequence visualization, PAM wheels were constructed according to Leenay et al. using KronaExcelTemplate (https://github.com/marbl/Krona/releases) (61). For each individual PAM sequence, a depletion score was estimated as the ratio of the normalized read count in output PAM libraries to the normalized read count in the control. In cases where PAM happened to be enriched in the “after the conjugation” library, the depletion score was changed to zero. The depletion scores were then used as the input for the Krona plot (61).

10.1128/mBio.02136-21.9 TABLE S3 (A) Sequencing read counts for 630Δ*erm* PAM libraries before and after conjugation. (B) Sequencing read counts for R20291 PAM libraries before and after conjugation. Download Table S3, PDF file, 0.09 MB. Copyright © 2021 Maikova et al. 2021 Maikova et al. https://creativecommons.org/licenses/by/4.0/ This content is distributed under the terms of the Creative Commons Attribution 4.0 International license.

### Plasmid conjugation efficiency assays.

To evaluate the conjugation efficiency, conjugative plasmids carrying PAM-protospacer were transformed into the E. coli HB101 (RP4) strain and transferred to the C. difficile 630Δ*erm* or C. difficile R20291 strain by conjugation. The ratio of C. difficile transconjugants to the total number of CFUs was estimated by subculturing conjugation mixtures on BHI agar supplemented with Tm, Cs, and Cfx and comparing the CFU to the number of CFUs obtained after plating serial dilutions on BHI agar plates containing Cfx only.

### CRISPR adaptation assay and high-throughput sequencing of newly acquired spacers.

After overnight growth in BHI medium supplemented with Tm and ATc, pCas1-2-4-containing cells were twice transferred to BHI medium supplemented with ATc without Tm (I and II reseeding) (Fig. 4A). These additional steps were necessary to enrich the bacterial culture with cells that acquired new spacers. After each reseeding, two rounds of PCR were performed to detect spacer acquisition. For amplification, we used a specific set of primers for each array. Forward primers annealed to leader regions of CRISPR arrays and reverse primers annealed to the first or the second spacer (CRISPR 10 array) of native CRISPR arrays (Fig. 4A). Primers are listed in the Table S2.

PCR products corresponding to expanded CRISPR arrays were extracted from the gel and used for nested PCR with primers containing Illumina adapters for further high-throughput sequencing and bioinformatic analysis. The amplicons were sequenced using the Illumina NextSeq 500 system with 2-million-read coverage. Sequence reads were analyzed in R using ShortRead and Biostrings packages (62) as described previously (63, 64). Graphical representation of results was performed using ggplot2 package (65) and the EasyVisio tool, developed by E. Rubtsova.

Newly acquired spacers of 10 to 79 bp in length were mapped to the reference genomes of Clostridium difficile 630 (NCBI reference sequence NC_009089.1), pCD630 (NCBI reference sequence NC_008226.2), and pCas1-2-4, with one mismatch allowed. Three nucleotides upstream of the first protospacer position were considered a PAM sequence. Spacers that aligned to multiple positions within the same molecule were removed from the analysis. Spacers that aligned to a single DNA molecule were referred to as “unique,” and spacers that aligned to several molecules (but to a single position within each molecule) were referred to as “nonunique” and analyzed separately (Table S4). “Shifters” and “flippers” were removed from analysis (66).

In total, for CRISPR 8 and CRISPR 9, we found 5,077 spacers that mapped to 1,380 individual genomic protospacer positions (Table S4). One percent of all positions (14 protospacers) contributed most to sequenced spacers and corresponded to 27% of sequenced genomic spacers. The genomic coordinates of these seemingly “hot” protospacers were different for CRISPR 8 and CRISPR 9; therefore, it is unlikely that these positions represent true “hot” protospacers. We assumed that these seemingly “hot” protospacers could arise due to early acquisition of corresponding spacers followed by their spread in the population during prolonged cultivation. Alternatively, they could be the result of heterogeneity in amplification during two subsequent rounds of PCR. To avoid unwanted biases caused by these seemingly “hot” protospacers, we removed them from subsequent analyses of spacer lengths, protospacer distributions along the genome, and frequencies of associated PAM motifs.

### RNA extraction and qRT-PCR.

Total RNA was isolated from the C. difficile 630Δ*erm* strain after 4, 6, and 10 h of growth in tryptone-yeast extract (TY) medium corresponding to early exponential, late exponential, and stationary phases, respectively, as previously described (67). cDNA synthesis by reverse transcription and quantitative real-time PCR analysis was performed as previously described (68) using a Bio-Rad CFX Connect real-time system. The expression levels of CRISPR arrays were calculated relative to that of the 16S RNA gene (69).

### Data availability.

Raw sequencing data have been deposited in the National Center for Biotechnology Information Sequence Read Archive under BioProject identifier (ID) PRJNA719030.

## References

[1] Oren A, Rupnik M. 2018. *Clostridium difficile* and *Clostridioides difficile*: two validly published and correct names. Anaerobe 52:125–126. doi:10.1016/j.anaerobe.2018.07.005. 30031828

[2] Warny M, Pepin J, Fang A, Killgore G, Thompson A, Brazier J, Frost E, McDonald LC. 2005. Toxin production by an emerging strain of *Clostridium difficile* associated with outbreaks of severe disease in North America and Europe. Lancet 366:1079–1084. doi:10.1016/S0140-6736(05)67420-X. 16182895

[3] Banawas SS. 2018. *Clostridium difficile* infections: a global overview of drug sensitivity and resistance mechanisms. Biomed Res Int 2018:8414257–8414259. doi:10.1155/2018/8414257. 29682562 PMC5841113

[4] Coignard B, Barbut F, Blanckaert K, Thiolet JM, Poujol I, Carbonne A, Petit JC, Desenclos JC. 2006. Emergence of *Clostridium difficile* toxinotype III, PCR-ribotype 027-associated disease, France, 2006. Euro Surveill 11:E060914.1. doi:10.2807/esw.11.37.03044-en. 17075146

[5] Abt MC, McKenney PT, Pamer EG. 2016. *Clostridium difficile* colitis: pathogenesis and host defence. Nat Rev Microbiol 14:609–620. doi:10.1038/nrmicro.2016.108. 27573580 PMC5109054

[6] Lim SC, Knight DR, Riley TV. 2020. *Clostridium difficile* and one health. Clin Microbiol Infect 26:857–863. doi:10.1016/j.cmi.2019.10.023. 31682985

[7] Smits WK, Lyras D, Lacy DB, Wilcox MH, Kuijper EJ. 2016. *Clostridium difficile* infection. Nat Rev Dis Primers 2:16020. doi:10.1038/nrdp.2016.20. 27158839 PMC5453186

[8] Carroll KC, Bartlett JG. 2011. Biology of *Clostridium difficile*: implications for epidemiology and diagnosis. Annu Rev Microbiol 65:501–521. doi:10.1146/annurev-micro-090110-102824. 21682645

[9] Just I, Selzer J, Wilm M, von Eichel-Streiber C, Mann M, Aktories K. 1995. Glucosylation of Rho proteins by *Clostridium difficile* toxin B. Nature 375:500–503. doi:10.1038/375500a0. 7777059

[10] Vedantam G, Clark A, Chu M, McQuade R, Mallozzi M, Viswanathan VK. 2012. *Clostridium difficile* infection: toxins and non-toxin virulence factors, and their contributions to disease establishment and host response. Gut Microbes 3:121–134. doi:10.4161/gmic.19399. 22555464 PMC3370945

[11] Rupnik M, Wilcox MH, Gerding DN. 2009. *Clostridium difficile* infection: new developments in epidemiology and pathogenesis. Nat Rev Microbiol 7:526–536. doi:10.1038/nrmicro2164. 19528959

[12] Ðapa T, Dapa T, Leuzzi R, Ng YK, Baban ST, Adamo R, Kuehne SA, Scarselli M, Minton NP, Serruto D, Unnikrishnan M. 2013. Multiple factors modulate biofilm formation by the anaerobic pathogen *Clostridium difficile*. J Bacteriol 195:545–555. doi:10.1128/JB.01980-12. 23175653 PMC3554014

[13] Nale JY, Chutia M, Carr P, Hickenbotham PT, Clokie MRJ. 2016. ‘Get in early’; biofilm and wax moth (*Galleria mellonella*) models reveal new insights into the therapeutic potential of *Clostridium difficile* bacteriophages. Front Microbiol 7:1383. doi:10.3389/fmicb.2016.01383. 27630633 PMC5005339

[14] Soavelomandroso AP, Gaudin F, Hoys S, Nicolas V, Vedantam G, Janoir C, Bouttier S. 2017. Biofilm structures in a mono-associated mouse model of *Clostridium difficile* infection. Front Microbiol 8:2086. doi:10.3389/fmicb.2017.02086. 29118745 PMC5661025

[15] Mick E, Stern A, Sorek R. 2013. Holding a grudge: persisting anti-phage CRISPR immunity in multiple human gut microbiomes. RNA Biol 10:900–906. doi:10.4161/rna.23929. 23439321 PMC3737347

[16] Stern A, Mick E, Tirosh I, Sagy O, Sorek R. 2012. CRISPR targeting reveals a reservoir of common phages associated with the human gut microbiome. Genome Res 22:1985–1994. doi:10.1101/gr.138297.112. 22732228 PMC3460193

[17] Sorek R, Lawrence CM, Wiedenheft B. 2013. CRISPR-mediated adaptive immune systems in bacteria and archaea. Annu Rev Biochem 82:237–266. doi:10.1146/annurev-biochem-072911-172315. 23495939

[18] Grissa I, Vergnaud G, Pourcel C. 2007. The CRISPRdb database and tools to display CRISPRs and to generate dictionaries of spacers and repeats. BMC Bioinformatics 8:172. doi:10.1186/1471-2105-8-172. 17521438 PMC1892036

[19] Wright AV, Wang JY, Burstein D, Harrington LB, Paez-Espino D, Kyrpides NC, Iavarone AT, Banfield JF, Doudna JA. 2019. A functional mini-integrase in a two-protein-type V-C CRISPR system. Mol Cell 73:727–737.e3. doi:10.1016/j.molcel.2018.12.015. 30709710 PMC6386590

[20] Shmakov SA, Sitnik V, Makarova KS, Wolf YI, Severinov KV, Koonin EV. 2017. The CRISPR spacer space is dominated by sequences from species-specific mobilomes. mBio 8:e01397-17. doi:10.1128/mBio.01397-17. 28928211 PMC5605939

[21] Yosef I, Goren MG, Qimron U. 2012. Proteins and DNA elements essential for the CRISPR adaptation process in *Escherichia coli*. Nucleic Acids Res 40:5569–5576. doi:10.1093/nar/gks216. 22402487 PMC3384332

[22] Wang R, Li M, Gong L, Hu S, Xiang H. 2016. DNA motifs determining the accuracy of repeat duplication during CRISPR adaptation in *Haloarcula hispanica*. Nucleic Acids Res 44:4266–4277. doi:10.1093/nar/gkw260. 27085805 PMC4872114

[23] Wei Y, Chesne MT, Terns RM, Terns MP. 2015. Sequences spanning the leader-repeat junction mediate CRISPR adaptation to phage in *Streptococcus thermophilus*. Nucleic Acids Res 43:1749–1758. doi:10.1093/nar/gku1407. 25589547 PMC4330368

[24] Marraffini LA. 2015. CRISPR-Cas immunity in prokaryotes. Nature 526:55–61. doi:10.1038/nature15386. 26432244

[25] Garneau JE, Dupuis M-V, Villion M, Romero DA, Barrangou R, Boyaval P, Fremaux C, Horvath P, Magadán AH, Moineau S. 2010. The CRISPR/Cas bacterial immune system cleaves bacteriophage and plasmid DNA. Nature 468:67–71. doi:10.1038/nature09523. 21048762

[26] Koonin EV, Makarova KS, Zhang F. 2017. Diversity, classification and evolution of CRISPR-Cas systems. Curr Opin Microbiol 37:67–78. doi:10.1016/j.mib.2017.05.008. 28605718 PMC5776717

[27] Amitai G, Sorek R. 2016. CRISPR–Cas adaptation: insights into the mechanism of action. Nat Rev Microbiol 14:67–76. doi:10.1038/nrmicro.2015.14. 26751509

[28] Lee H, Zhou Y, Taylor DW, Sashital DG. 2018. Cas4-dependent prespacer processing ensures high-fidelity programming of CRISPR arrays. Mol Cell 70:48.e5–59.e5. doi:10.1016/j.molcel.2018.03.003. 29602742 PMC5889325

[29] Kieper SN, Almendros C, Behler J, McKenzie RE, Nobrega FL, Haagsma AC, Vink JNA, Hess WR, Brouns SJJ. 2018. Cas4 facilitates PAM-compatible spacer selection during CRISPR adaptation. Cell Rep 22:3377–3384. doi:10.1016/j.celrep.2018.02.103. 29590607 PMC5896167

[30] Lee H, Dhingra Y, Sashital DG. 2019. The Cas4-Cas1-Cas2 complex mediates precise prespacer processing during CRISPR adaptation. Elife 8:e44248. doi:10.7554/eLife.44248. 31021314 PMC6519985

[31] Zhang Z, Pan S, Liu T, Li Y, Peng N. 2019. Cas4 nucleases can effect specific integration of CRISPR spacers. J Bacteriol 201:e00747-18. doi:10.1128/JB.00747-18. 30936372 PMC6531622

[32] Westra ER, van Erp PBG, Künne T, Wong SP, Staals RHJ, Seegers CLC, Bollen S, Jore MM, Semenova E, Severinov K, de Vos WM, Dame RT, de Vries R, Brouns SJJ, van der Oost J. 2012. CRISPR immunity relies on the consecutive binding and degradation of negatively supercoiled invader DNA by Cascade and Cas3. Mol Cell 46:595–605. doi:10.1016/j.molcel.2012.03.018. 22521689 PMC3372689

[33] Xue C, Seetharam AS, Musharova O, Severinov K, Brouns SJJ, Severin AJ, Sashital DG. 2015. CRISPR interference and priming varies with individual spacer sequences. Nucleic Acids Res 43:10831–10847. doi:10.1093/nar/gkv1259. 26586800 PMC4678831

[34] Xue C, Whitis NR, Sashital DG. 2016. Conformational control of Cascade interference and priming activities in CRISPR immunity. Mol Cell 64:826–834. doi:10.1016/j.molcel.2016.09.033. 27871367 PMC5561731

[35] Makarova KS, Haft DH, Barrangou R, Brouns SJJ, Charpentier E, Horvath P, Moineau S, Mojica FJM, Wolf YI, Yakunin AF, van der Oost J, Koonin EV. 2011. Evolution and classification of the CRISPR–Cas systems. Nat Rev Microbiol 9:467–477. doi:10.1038/nrmicro2577. 21552286 PMC3380444

[36] Takeuchi N, Wolf YI, Makarova KS, Koonin EV. 2012. Nature and intensity of selection pressure on CRISPR-associated genes. J Bacteriol 194:1216–1225. doi:10.1128/JB.06521-11. 22178975 PMC3294813

[37] Soutourina O, Monot M, Boudry P, Saujet L, Pichon C, Sismeiro O, Semenova E, Severinov K, Le Bouguenec C, Coppée JY, Dupuy B, Martin-Verstraete I. 2013. Genome-wide identification of regulatory RNAs in the human pathogen *Clostridium difficile*. PLoS Genet 9:e1003493. doi:10.1371/journal.pgen.1003493. 23675309 PMC3649979

[38] Boudry P, Semenova E, Monot M, Datsenko KA, Lopatina A, Sekulovic O, Ospina-Bedoya M, Fortier LC, Severinov K, Dupuy B, Soutourina O. 2015. Function of the CRISPR-Cas system of the human pathogen *Clostridium difficile*. mBio 6:e01112-15. doi:10.1128/mBio.01112-15. 26330515 PMC4556805

[39] Andersen JM, Shoup M, Robinson C, Britton R, Olsen KEP, Barrangou R. 2016. CRISPR diversity and microevolution in *Clostridium difficile*. Genome Biol Evol 8:2841–2855. doi:10.1093/gbe/evw203. 27576538 PMC5630864

[40] Hargreaves KR, Flores CO, Lawley TD, Clokie RJ, Trevor D, Hargreaves KR, Flores CO, Lawley TD, Clokie RJ. 2014. Abundant and diverse clustered regularly interspaced short palindromic repeat spacers in *Clostridium difficile* strains and prophages target multiple phage types within this pathogen. mBio 5:e01045-13. doi:10.1128/mBio.01045-13. 25161187 PMC4173771

[41] Maikova A, Kreis V, Boutserin A, Severinov K, Soutourina O. 2019. Using an endogenous CRISPR-Cas system for genome editing in the human pathogen *Clostridium difficile*. Appl Environ Microbiol 85:e01416-19. doi:10.1128/AEM.01416-19. 31399410 PMC6805081

[42] Semenova E, Jore MM, Datsenko KA, Semenova A, Westra ER, Wanner B, Van der Oost J, Brouns SJJ, Severinov K. 2011. Interference by clustered regularly interspaced short palindromic repeat (CRISPR) RNA is governed by a seed sequence. Proc Natl Acad Sci USA 108:10098–10103. doi:10.1073/pnas.1104144108. 21646539 PMC3121866

[43] Sebaihia M, Wren BW, Mullany P, Fairweather NF, Minton N, Stabler R, Thomson NR, Roberts AP, Cerdeño-Tárraga AM, Wang H, Holden MTG, Wright A, Churcher C, Quail MA, Baker S, Bason N, Brooks K, Chillingworth T, Cronin A, Davis P, Dowd L, Fraser A, Feltwell T, Hance Z, Holroyd S, Jagels K, Moule S, Mungall K, Price C, Rabbinowitsch E, Sharp S, Simmonds M, Stevens K, Unwin L, Whithead S, Dupuy B, Dougan G, Barrell B, Parkhill J. 2006. The multidrug-resistant human pathogen *Clostridium difficile* has a highly mobile, mosaic genome. Nat Genet 38:779–786. doi:10.1038/ng1830. 16804543

[44] Levy A, Goren MG, Yosef I, Auster O, Manor M, Amitai G, Edgar R, Qimron U, Sorek R. 2015. CRISPR adaptation biases explain preference for acquisition of foreign DNA. Nature 520:505–510. doi:10.1038/nature14302. 25874675 PMC4561520

[45] Stachler AE, Wörtz J, Alkhnbashi OS, Turgeman-Grott I, Smith R, Allers T, Backofen R, Gophna U, Marchfelder A. 2020. Adaptation induced by self-targeting in a type I-B CRISPR-Cas system. J Biol Chem 295:13502–13515. doi:10.1074/jbc.RA120.014030. 32723866 PMC7521656

[46] Shiimori M, Garrett SC, Chambers DP, Glover CVC, Graveley BR, Terns MP. 2017. Role of free DNA ends and protospacer adjacent motifs for CRISPR DNA uptake in *Pyrococcus furiosus*. Nucleic Acids Res 45:11281–11294. doi:10.1093/nar/gkx839. 29036456 PMC5737086

[47] Shah SA, Erdmann S, Mojica FJM, Garrett RA. 2013. Protospacer recognition motifs. RNA Biol 10:891–899. doi:10.4161/rna.23764. 23403393 PMC3737346

[48] Fischer S, Maier LK, Stoll B, Brendel J, Fischer E, Pfeiffer F, Dyall-Smith M, Marchfelder A. 2012. An archaeal immune system can detect multiple protospacer adjacent motifs (PAMs) to target invader DNA. J Biol Chem 287:33351–33365. doi:10.1074/jbc.M112.377002. 22767603 PMC3460438

[49] Stoll B, Maier L-K, Lange SJ, Brendel J, Fischer S, Backofen R, Marchfelder A. 2013. Requirements for a successful defence reaction by the CRISPR–Cas subtype I-B system. Biochem Soc Trans 41:1444–1448. doi:10.1042/BST20130098. 24256235

[50] Maier L-K, Stachler A-E, Brendel J, Stoll B, Fischer S, Haas K, Schwarz T, Alkhnbashi OS, Sharma K, Urlaub H, Backofen R, Gophna U, Marchfelder A. 2019. The nuts and bolts of the *Haloferax* CRISPR-Cas system I-B. RNA Biol 16:469–480. doi:10.1080/15476286.2018.1460994. 29649958 PMC6546412

[51] Garrett S, Shiimori M, Watts EA, Clark L, Graveley BR, Terns MP. 2020. Primed CRISPR DNA uptake in *Pyrococcus furiosus*. Nucleic Acids Res 48:6120–6135. doi:10.1093/nar/gkaa381. 32421777 PMC7293040

[52] Turgeman-Grott I, Joseph S, Marton S, Eizenshtein K, Naor A, Soucy SM, Stachler AE, Shalev Y, Zarkor M, Reshef L, Altman-Price N, Marchfelder A, Gophna U. 2019. Pervasive acquisition of CRISPR memory driven by inter-species mating of archaea can limit gene transfer and influence speciation. Nat Microbiol 4:177–186. doi:10.1038/s41564-018-0302-8. 30478289 PMC6298592

[53] Li M, Wang R, Zhao D, Xiang H. 2014. Adaptation of the *Haloarcula hispanica* CRISPR-Cas system to a purified virus strictly requires a priming process. Nucleic Acids Res 42:2483–2492. doi:10.1093/nar/gkt1154. 24265226 PMC3936756

[54] Maikova A, Peltier J, Boudry P, Hajnsdorf E, Kint N, Monot M, Poquet I, Martin-Verstraete I, Dupuy B, Soutourina O. 2018. Discovery of new type I toxin–antitoxin systems adjacent to CRISPR arrays in *Clostridium difficile*. Nucleic Acids Res 46:4733–4751. doi:10.1093/nar/gky124. 29529286 PMC5961336

[55] Peltier J, Hamiot A, Garneau JR, Boudry P, Maikova A, Hajnsdorf E, Fortier LC, Dupuy B, Soutourina O. 2020. Type I toxin-antitoxin systems contribute to the maintenance of mobile genetic elements in *Clostridioides difficile*. Commun Biol 3:718. doi:10.1038/s42003-020-01448-5. 33247281 PMC7699646

[56] Bertani G. 1951. Studies on lysogenesis. I. The mode of phage liberation by lysogenic *Escherichia coli*. J Bacteriol 62:293–300. doi:10.1128/jb.62.3.293-300.1951. 14888646 PMC386127

[57] Fagan RP, Fairweather NF. 2011. *Clostridium difficile* has two parallel and essential Sec secretion systems. J Biol Chem 286:27483–27493. doi:10.1074/jbc.M111.263889. 21659510 PMC3149341

[58] Gibson DG, Young L, Chuang R-Y, Venter JC, Hutchison CA, Smith HO. 2009. Enzymatic assembly of DNA molecules up to several hundred kilobases. Nat Methods 6:343–345. doi:10.1038/nmeth.1318. 19363495

[59] Li H, Durbin R. 2009. Fast and accurate short read alignment with Burrows-Wheeler transform. Bioinformatics 25:1754–1760. doi:10.1093/bioinformatics/btp324. 19451168 PMC2705234

[60] Crooks GE, Hon G, Chandonia J-M, Brenner SE. 2004. WebLogo: a sequence logo generator. Genome Res 14:1188–1190. doi:10.1101/gr.849004. 15173120 PMC419797

[61] Leenay RT, Maksimchuk KR, Slotkowski RA, Agrawal RN, Gomaa AA, Briner AE, Barrangou R, Beisel CL. 2016. Identifying and visualizing functional PAM diversity across CRISPR-Cas systems. Mol Cell 62:137–147. doi:10.1016/j.molcel.2016.02.031. 27041224 PMC4826307

[62] Morgan M, Anders S, Lawrence M, Aboyoun P, Pagès H, Gentleman R. 2009. ShortRead: a bioconductor package for input, quality assessment and exploration of high-throughput sequence data. Bioinformatics 25:2607–2608. doi:10.1093/bioinformatics/btp450. 19654119 PMC2752612

[63] Musharova O, Vyhovskyi D, Medvedeva S, Guzina J, Zhitnyuk Y, Djordjevic M, Severinov K, Savitskaya E. 2018. Avoidance of trinucleotide corresponding to consensus protospacer adjacent motif controls the efficiency of prespacer selection during primed adaptation. mBio 9:e02169-18. doi:10.1128/mBio.02169-18. 30514784 PMC6282206

[64] Shiriaeva AA, Savitskaya E, Datsenko KA, Vvedenskaya IO, Fedorova I, Morozova N, Metlitskaya A, Sabantsev A, Nickels BE, Severinov K, Semenova E. 2019. Detection of spacer precursors formed in vivo during primed CRISPR adaptation. Nat Commun 10:4603. doi:10.1038/s41467-019-12417-w. 31601800 PMC6787059

[65] Wickham H. 2009. ggplot2: elegant graphics for data analysis. Springer, Berlin, Germany.

[66] Shmakov S, Savitskaya E, Semenova E, Logacheva MD, Datsenko KA, Severinov K. 2014. Pervasive generation of oppositely oriented spacers during CRISPR adaptation. Nucleic Acids Res 42:5907–5916. doi:10.1093/nar/gku226. 24728991 PMC4027179

[67] André G, Even S, Putzer H, Burguière P, Croux C, Danchin A, Martin-Verstraete I, Soutourina O. 2008. S-box and T-box riboswitches and antisense RNA control a sulfur metabolic operon of *Clostridium acetobutylicum*. Nucleic Acids Res 36:5955–5969. doi:10.1093/nar/gkn601. 18812398 PMC2566862

[68] Saujet L, Monot M, Dupuy B, Soutourina O, Martin-Verstraete I. 2011. The key sigma factor of transition phase, sigh, controls sporulation, metabolism, and virulence factor expression in *Clostridium difficile*. J Bacteriol 193:3186–3196. doi:10.1128/JB.00272-11. 21572003 PMC3133256

[69] Metcalf D, Sharif S, Weese JS. 2010. Evaluation of candidate reference genes in *Clostridium difficile* for gene expression normalization. Anaerobe 16:439–443. doi:10.1016/j.anaerobe.2010.06.007. 20599622

[70] Oliveira Paiva AM, van Eijk E, Friggen AH, Weigel C, Smits WK. 2020. Identification of the unwinding region in the *Clostridioides difficile* chromosomal origin of replication. Front Microbiol 11:581401. doi:10.3389/fmicb.2020.581401. 33133049 PMC7561715

