# Supplementary figures and images for: Protospacer-Adjacent Motif Specificity during Clostridioides difficile Type I-B CRISPR-Cas Interference and Adaptation
Source: mBio. 2021 Aug 24;12(4):e02136-21. doi: 10.1128/mBio.02136-21 (PMC8406132; doi:10.1128/mBio.02136-21)

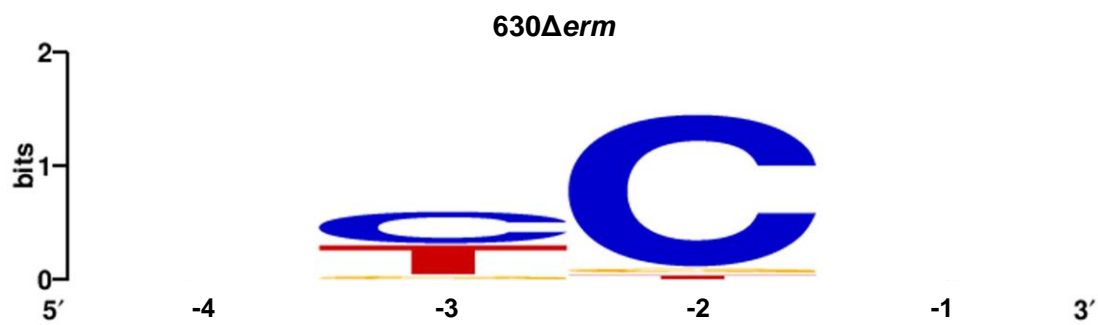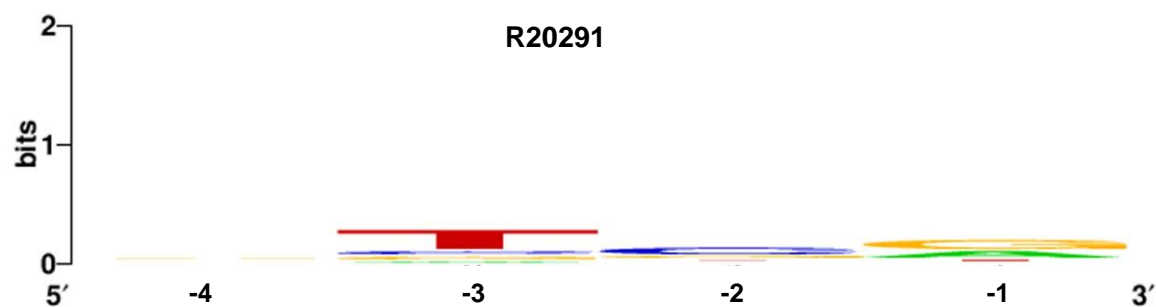

**Fig. S1**

Supplement: FIG S1 [file mbio.02136-21-sf001.pdf]

**A**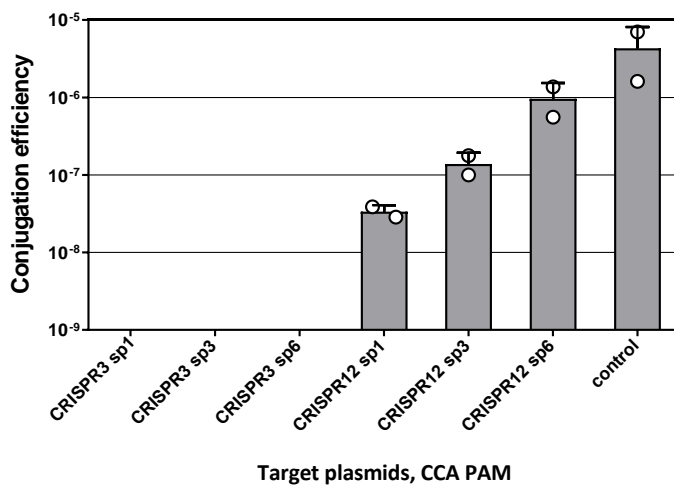**B**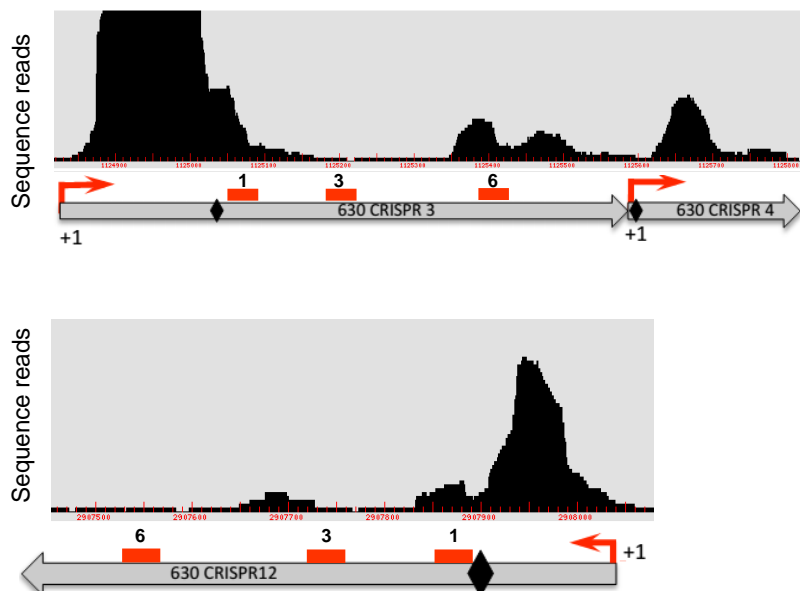**Fig. S2**

Supplement: FIG S2 [file mbio.02136-21-sf002.pdf]

**A**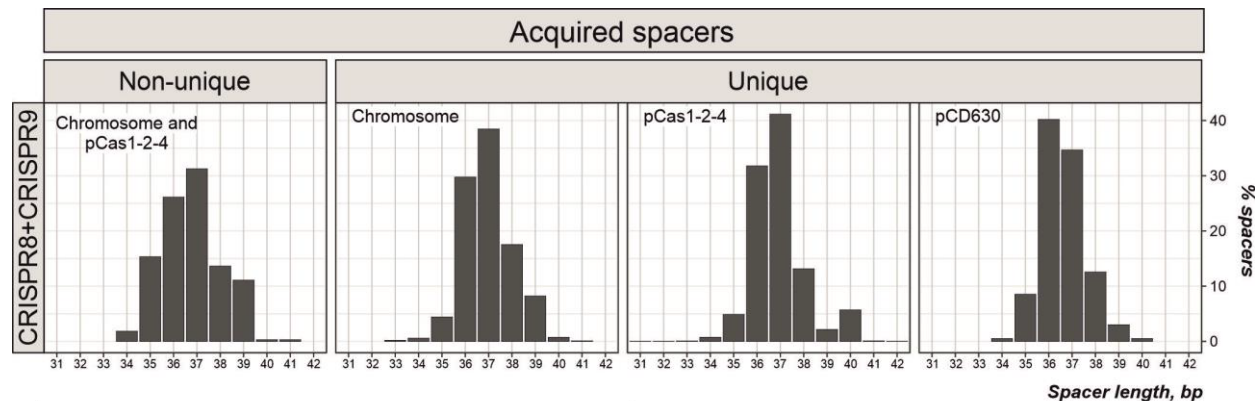**B**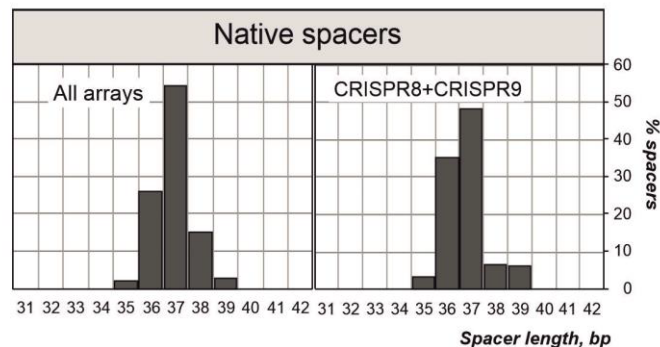**Fig. S3**

Supplement: FIG S3 [file mbio.02136-21-sf003.pdf]

A

## CRISPR8+9

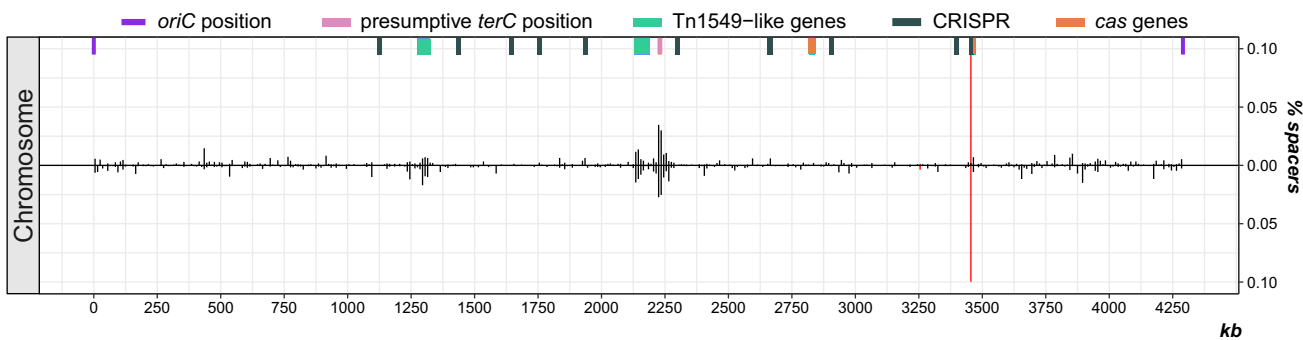

B

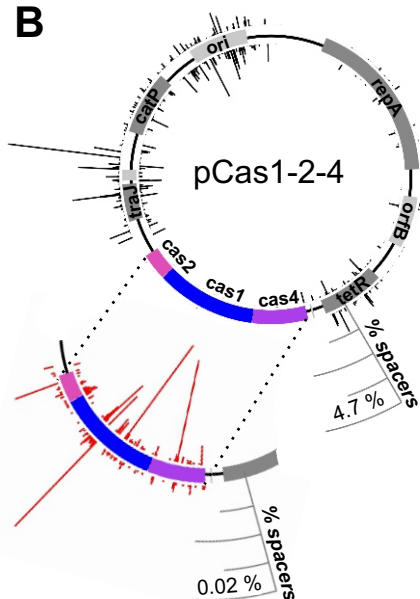

C

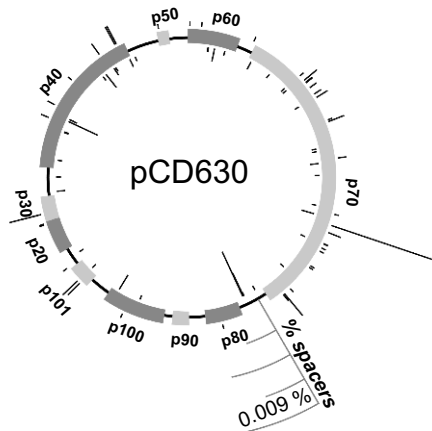

Fig. S4

Supplement: FIG S4 [file mbio.02136-21-sf004.pdf]

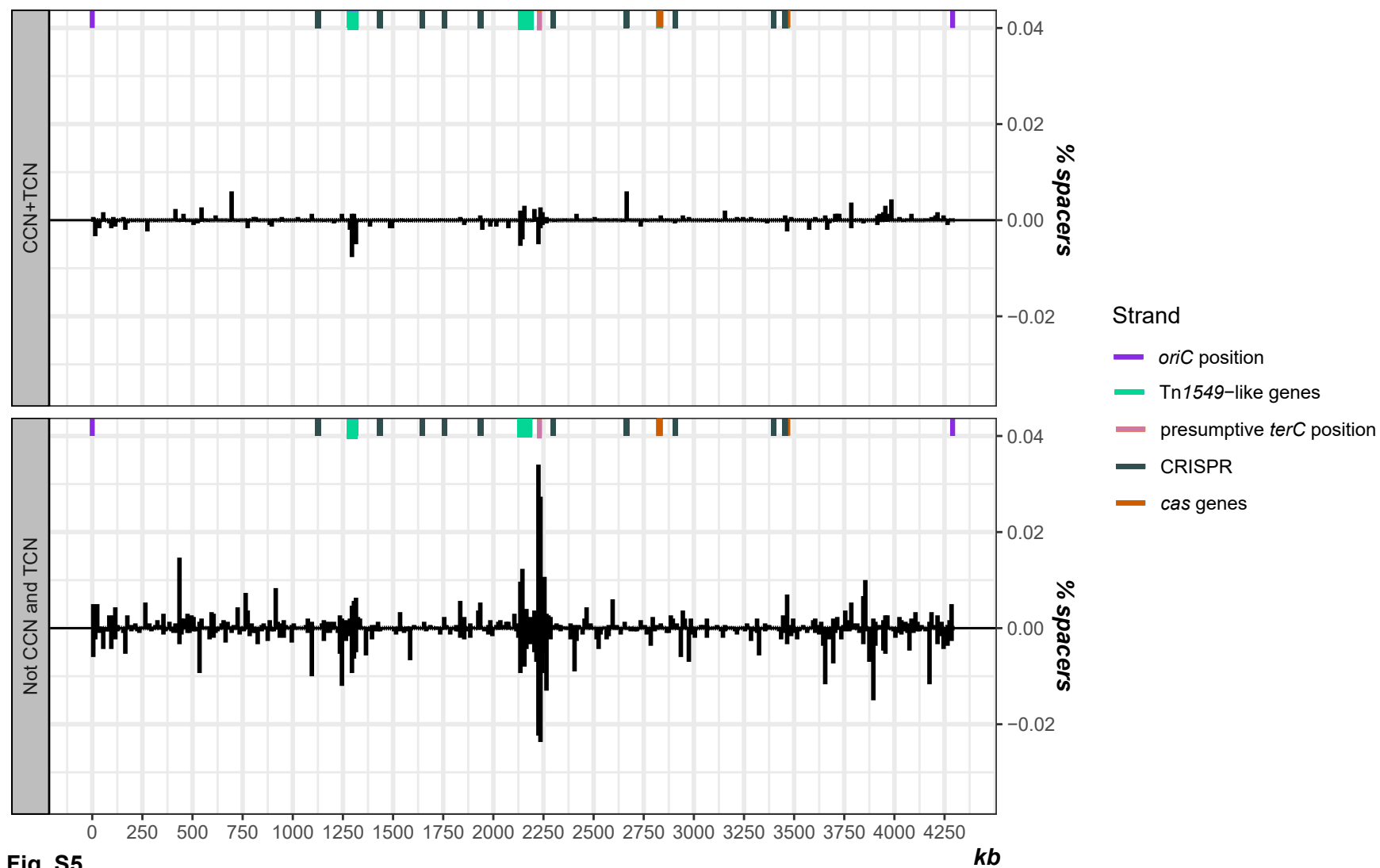

Fig. S5

Supplement: FIG S5 [file mbio.02136-21-sf005.pdf]

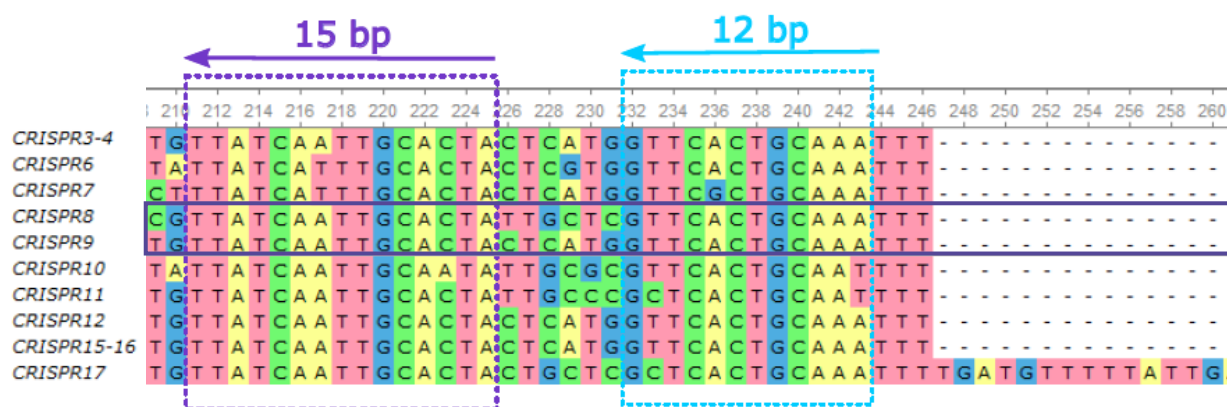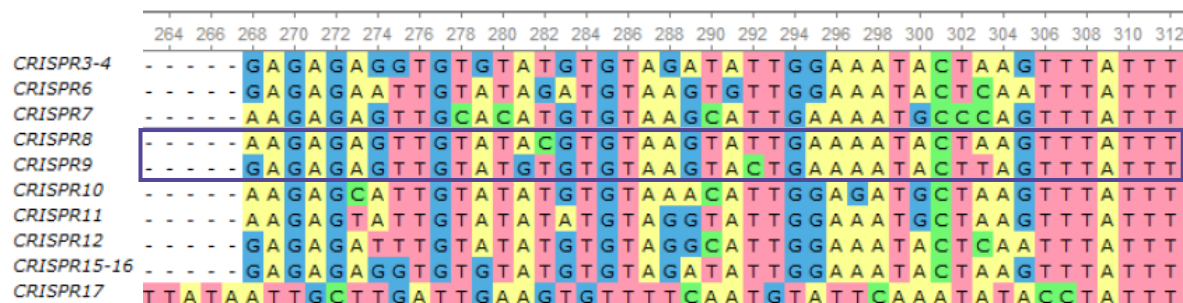

CRISPR repeat

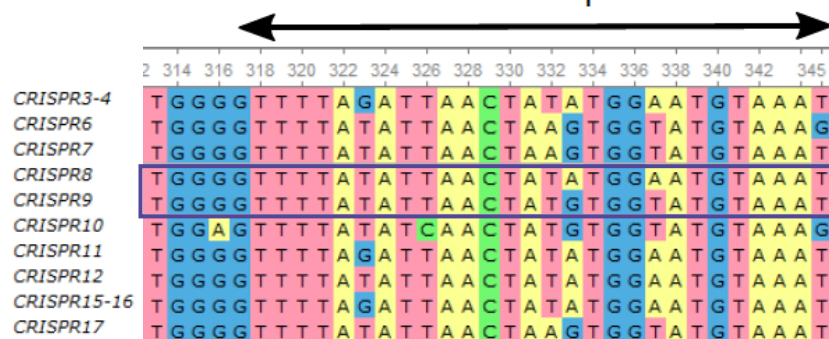

Fig. S6

Supplement: FIG S6 [file mbio.02136-21-sf006.pdf]
