# Supplementary material for: Protospacer-Adjacent Motif Specificity during Clostridioides difficile Type I-B CRISPR-Cas Interference and Adaptation
Source: mBio. 2021 Aug 24;12(4):e02136-21. doi: 10.1128/mBio.02136-21 (PMC8406132; doi:10.1128/mBio.02136-21)
Supplement: TABLE S1 [file mbio.02136-21-st001.pdf]

Table S1. Strains and plasmids used in this study

**Strain**	**Genotype**	**Source**
E. coli		
NEB-10 beta	*Δ(ara-leu) 7697 araD139 fhuA ΔlacX74 galK16 galE15 e14- ϕ80dlacZΔM15 recA1 relA1 endA1 nupG rpsL (StrR) rph spoT1 Δ(mrrhsdRMS-mcrBC)*	New England Biolabs
HB101 (RP4)	*supE44 aa14 galK2 lacY1 Δ(gpt-proA) 62 rpsL20 (StrR)xyl-5 mtl-1 recA13 Δ(mcrC-mrr) hsdSB (rB-mB-)* RP4 *(Tra+ IncP ApR KmR TcR)*	Laboratory stock
C. difficile		
630Δ*erm*	Sequenced reference strain Δ*ermB*	Laboratory stock (1)
R20291	PCR-ribotype 027 epidemic strain	Laboratory stock
CNRS_CD059	630Δ*erm* carrying pRPFΔ*gus*	This work
CNRS_CD001	630Δ*erm* carrying pCas1-2 plasmid	This work
CNRS_CD002	630Δ*erm* carrying pCas1-2-4 plasmid	This work
**Plasmid**	**Description**	**Reference**
pRPF185Δ*gus*	P_{tet}-*gusA* Tm^R expression and cloning *Clostridium-Escherichia coli* shuttle vector, pRPF185 vector derivative	(2, 3)
pDIA6435	pRPF185Δ*gus* with the 5' CCA-PAM protospacer, corresponding to the spacer1 from 630Δ*erm* CRISPR3 array	This work
pDIA6436	pRPF185Δ*gus* with the 5' CCA-PAM protospacer, corresponding to the spacer1 from 630Δ*erm* CRISPR4 array	This work
pDIA6437	pRPF185Δ*gus* with the 5' CCA-PAM protospacer, corresponding to the spacer1 from 630Δ*erm* CRISPR6 array	This work
pDIA6438	pRPF185Δ*gus* with the 5' CCA-PAM protospacer, corresponding to the spacer1 from 630Δ*erm* CRISPR7 array	This work
pDIA6439	pRPF185Δ*gus* with the 5' CCA-PAM protospacer, corresponding to the spacer1 from 630Δ*erm* CRISPR8 array	This work
pDIA6440	pRPF185Δ*gus* with the 5' CCA-PAM protospacer, corresponding to the spacer1 from 630Δ*erm* CRISPR9 array	This work
pDIA6441	pRPF185Δ*gus* with the 5' CCA-PAM protospacer, corresponding to the spacer1 from 630Δ*erm* CRISPR10 array	This work
pDIA6442	pRPF185Δ*gus* with the 5' CCA-PAM protospacer, corresponding to the spacer1 from 630Δ*erm* CRISPR11 array	This work

pDIA6443	pRPF185Δ*gus* with the 5' CCA-PAM protospacer, corresponding to the spacer1 from 630Δ*erm* CRISPR12 array	This work
pDIA6444	pRPF185Δ*gus* with the 5' CCA-PAM protospacer, corresponding to the spacer1 from 630Δ*erm* CRISPR17 array	This work
pDIA6445	pRPF185Δ*gus* with the 5' CCA-PAM protospacer, corresponding to the spacer3 from 630Δ*erm* CRISPR3 array	This work
pDIA6446	pRPF185Δ*gus* with the 5' CCA-PAM protospacer, corresponding to the spacer6 from 630Δ*erm* CRISPR3 array	This work
pDIA6447	pRPF185Δ*gus* with the 5' CCA-PAM protospacer, corresponding to the spacer3 from 630Δ*erm* CRISPR12 array	This work
pDIA6448	pRPF185Δ*gus* with the 5' CCA-PAM protospacer, corresponding to the spacer6 from 630Δ*erm* CRISPR12 array	This work
pDIA6475	pRPF185Δ*gus* with the 5' CCC-PAM protospacer, corresponding to the spacer1 from R20291 CRISPR13 array	This work
pDIA6476	pRPF185Δ*gus* with the 5' GAG-PAM protospacer, corresponding to the spacer1 from R20291 CRISPR13 array	This work
pDIA6477	pRPF185Δ*gus* with the 5' CCT-PAM protospacer, corresponding to the spacer1 from R20291 CRISPR13 array	This work
pDIA6478	pRPF185Δ*gus* with the 5' AAT-PAM protospacer, corresponding to the spacer1 from R20291 CRISPR13 array	This work
pDIA6479	pRPF185Δ*gus* with the 5' CCA-PAM protospacer with a mutation in the 1st position, corresponding to the spacer1 from R20291 CRISPR13 array	This work
pDIA6480	pRPF185Δ*gus* with the 5' CCA-PAM protospacer, corresponding to the spacer1 from R20291 CRISPR13 array	This work
pDIA6493	pRPF185Δ*gus* with the 5' CCG-PAM protospacer, corresponding to the spacer1 from R20291 CRISPR13 array	This work
CNRS_p045	pRPF185Δ*gus* with the 5' CCC-PAM protospacer, corresponding to the spacer1 from 630Δ*erm* CRISPR3 array	This work
CNRS_p046	pRPF185Δ*gus* with the 5' TCA-PAM protospacer, corresponding to the spacer1 from 630Δ*erm* CRISPR3 array	This work
CNRS_p047	pRPF185Δ*gus* with the 5' TCC-PAM protospacer, corresponding to the spacer1 from 630Δ*erm* CRISPR3 array	This work

CNRS_p048	pRPF185Δ*gus* with the 5' TCA-PAM protospacer, corresponding to the spacer1 from R20291 CRISPR13 array	This work
CNRS_p049	pRPF185Δ*gus* with the 5' CCG-PAM protospacer, corresponding to the spacer1 from 630Δ*erm* CRISPR3 array	This work
CNRS_p050	pRPF185Δ*gus* with the 5' TCT-PAM protospacer, corresponding to the spacer1 from 630Δ*erm* CRISPR3 array	This work
CNRS_p051	pRPF185Δ*gus* with the 5' TCG-PAM protospacer, corresponding to the spacer1 from 630Δ*erm* CRISPR3 array	This work
CNRS_p052	pRPF185Δ*gus* with the 5' TCT-PAM protospacer, corresponding to the spacer1 from R20291 CRISPR13 array	This work
CNRS_p053	pRPF185Δ*gus* with the 5' TCG-PAM protospacer, corresponding to the spacer1 from R20291 CRISPR13 array	This work
CNRS_p054	pRPF185Δ*gus* with the 5' TCC-PAM protospacer, corresponding to the spacer1 from R20291 CRISPR13 array	This work
pCas1-2	pRPF185Δ*gus* carrying 630Δ*erm cas1* and *cas2* genes under the control of P_{tet} promoter	This work
pCas1-2-4	pRPF185Δ*gus* carrying 630Δ*erm cas1*, *cas2* and *cas4* genes under the control of P_{tet} promoter	This work

1. Hussain,H.A., Roberts,A.P. and Mullany,P. (2005) Generation of an erythromycin-sensitive derivative of *Clostridium difficile* strain 630 (630Δ*erm*) and demonstration that the conjugative transposon Tn*916*ΔE enters the genome of this strain at multiple sites. *J. Med. Microbiol.*, **54**, 137–141.
2. Soutourina,O., Monot,M., Boudry,P., Saujet,L., Pichon,C., Sismeiro,O., Semenova,E., Severinov,K., Le Bouguenec,C., Coppée,J.Y., *et al.* (2013) Genome-wide identification of regulatory RNAs in the human pathogen *Clostridium difficile*. *PLoS Genet.*, **9**, e1003493.
3. Fagan,R.P. and Fairweather,N.F. (2011) *Clostridium difficile* has two parallel and essential Sec secretion systems. *J. Biol. Chem.*, **286**, 27483–27493.
