# Supplementary material for: Protospacer-Adjacent Motif Specificity during Clostridioides difficile Type I-B CRISPR-Cas Interference and Adaptation
Source: mBio. 2021 Aug 24;12(4):e02136-21. doi: 10.1128/mBio.02136-21 (PMC8406132; doi:10.1128/mBio.02136-21)
Supplement: TABLE S4 [file mbio.02136-21-st004.pdf]

Table S4. Number of spacers acquired into CRISPR8 and CRISPR9 arrays

Array	Reads, total	Spacers extracted	Unique alignment, number and % of spacers				Non-unique alignment, number and % of spacers				Total spacers aligned
			All DNAs	Chromosome	pCas1-2-4	pCD630	All DNAs	Chromosome	pCas1-2-4	pCD630	
**CRISPR8**	1805797	258588	220393	3303	216946	144	329	329	329	0	**220722**
			**99.85%**	**1.50%**	**98.29%**	**0.07%**	**0.15%**	**0.15%**	**0.15%**	**0.00%**	
**CRISPR9**	1711216	104508	78577	1774	76748	55	375	375	375	0	**78952**
			**99.53%**	**2.25%**	**97.21%**	**0.07%**	**0.47%**	**0.47%**	**0.47%**	**0.00%**	
**CRISPR8 and CRISPR9**	3517013	363096	298970	5077	293694	199	704	704	704	0	**299674**
			**99.77%**	**1.69%**	**98.00%**	**0.07%**	**0.23%**	**0.23%**	**0.23%**	**0.00%**	
