# Supplementary material for: Protospacer-Adjacent Motif Specificity during Clostridioides difficile Type I-B CRISPR-Cas Interference and Adaptation
Source: mBio. 2021 Aug 24;12(4):e02136-21. doi: 10.1128/mBio.02136-21 (PMC8406132; doi:10.1128/mBio.02136-21)
Supplement: TABLE S2 [file mbio.02136-21-st002.pdf]

Table S2. Oligonucleotides used in this study.

Name	Sequence (5'-3')	Description
**PAM libraries cloning into pRPF185Δ*gus* and analysis**		
IMV507	GGGATTTCTCACATAAAATAGAG	5'-pRPF185Δ*gus* insertion checking
IMV508	TAAAATAAGCTTGATCGTAGCG	3'-pRPF185Δ*gus* insertion checking
AM29	gcttgatcgtagcgttaacagatctgagctNNNNGTAGAGTCTTTATATGGTAGAGGTGGAATATATAAGTgatcctataagttttaataaaactttaaatag	630Δ*erm* PAM library 5'-NNNN-sp1CRISPR3/16
AM30	gcttgatcgtagcgttaacagatctgagctNNNNGTTGTAAGAAGTATCATTCTATTTTTTAATCTTTCTgatcctataagttttaataaaactttaaatag	R20291 PAM library 5'-NNNN-sp1CRISPR13
AM31	GCTTGATCGTAGCGTTAAC	PAM libraries double strand synthesis F
AM32	CTATTTAAAGTTTTATTAAAACTTATAGG	PAM libraries double strand synthesis R
AM245	tcgtcggcagcgtcagatgtgtataagagacagAGTATAATTAAAATAAGCTTGATCGT	PAM libraries Illumina F
AM246	gtctcgtgggctcggagatgtgtataagagacagAGAGAAGCCTTTTTCTATTTAAAG	PAM libraries Illumina R
**PAM-protospacers cloning into pRPF185Δ*gus***		
AM1	cCCAGTAGAGTCTTTATATGGTAGAGGTGGAATATATAAGTg	630Δ*erm* 5'-CCA-sp1CRISPR3/16-SacI
AM2	gatccACTTATATATTCCACCTCTACCATATAAAGACTCTACTGGgagct	630Δ*erm* 3'-CCA-sp1CRISPR3/16-BamHI
AM3	cCCAGTTTGAGAACGCTGTATAAAGCTTGTAGCTAGTTCTTg	630Δ*erm* 5'-CCA-sp1CRISPR4/15-SacI
AM4	gatccAAGAACTAGCTACAAGCTTTATACAGCGTTCTCAAACTGGgagct	630Δ*erm* 3'-CCA-sp1CRISPR4/15-BamHI
AM5	cCCAAAGCTTTCATAGCATCCTCTTCTGAACATTCATCACTg	630Δ*erm* 5'-CCA-sp1CRISPR6-SacI
AM6	gatccAGTGATGAATGTTCAGAAGAGGATGCTATGAAAGCTTTGGgagct	630Δ*erm* 3'-CCA-sp1CRISPR6-BamHI
AM7	cCCATAAAACCACTCAATTCTTAAAAGATACTGCAATTATTg	630Δ*erm* 5'-CCA-sp1CRISPR7-SacI
AM8	gatccAATAATTGCAGTATCTTTTAAGAATTGAGTGGTTTTATGGgagct	630Δ*erm* 3'-CCA-sp1CRISPR7-BamHI

AM9	cCCATATCTATTATTGGTATATTAAATGATTCTAATAATTCg	630Δ*erm* 5'-CCA-sp1CRISPR8-SacI
AM10	gatccGAATTATTAGAATCATTTAATATACCAATAATAGATATGGgagct	630Δ*erm* 3'-CCA-sp1CRISPR8-BamHI
AM11	cCCACTAGAATTAGAACTCATTATTAAAACCATTCTTGCAAGg	630Δ*erm* 5'-CCA-sp1CRISPR9-SacI
AM12	gatccCTTGCAAGAATGGTTTTAATAATGAGTTCTAATTCTAGTGGgagct	630Δ*erm* 3'-CCA-sp1CRISPR9-BamHI
AM13	cCCATTACTAAACATCTTATAACTTCTCTGAGAGCCTCTAGg	630Δ*erm* 5'-CCA-sp1CRISPR10-SacI
AM14	gatccCTAGAGGCTCTCAGAGAAGTTATAAGATGTTTAGTAATGGgagct	630Δ*erm* 3'-CCA-sp1CRISPR10-BamHI
AM15	cCCACTCCTTTCATTTCTCCTTTAGCTTCATAGCTTATTTTg	630Δ*erm* 5'-CCA-sp1CRISPR11-SacI
AM16	gatccAAAATAAGCTATGAAGCTAAAGGAGAAATGAAAGGAGTGGgagct	630Δ*erm* 3'-CCA-sp1CRISPR11-BamHI
AM17	cCCAAAAGGTGTCCATTGATTTCTTTCAGTTTCGGGAATAg	630Δ*erm* 5'-CCA-sp1CRISPR12-SacI
AM18	gatccTATTCCCGAAACTGAAAGAAATCAATGGACACCTTTTGGgagct	630Δ*erm* 3'-CCA-sp1CRISPR12-BamHI
AM19	cCCATTAGCTTCATAGCTTATTTTCTTTATTACTTCAATTTg	630Δ*erm* 5'-CCA-sp1CRISPR17-SacI
AM20	gatccAAATTGAAGTAATAAAGAAAATAAGCTATGAAGCTAATGGgagct	630Δ*erm* 3'-CCA-sp1CRISPR17-BamHI
AM21	cCCAACTGTATGCCATCTAAATGCATCATACAAACTTATTTg	630Δ*erm* 5'-CCA-sp3CRISPR3/16-SacI
AM22	gatccAAATAAGTTTGTATGATGCATTTAGATGGCATACAGTTGGgagct	630Δ*erm* 3'-CCA-sp3CRISPR3/16-BamHI
AM23	cCCAAATATAACAAAGTGGATGTTCTCTAAAAATAAAGAGGg	630Δ*erm* 5'-CCA-sp6CRISPR3/16-SacI
AM24	gatccCCTCTTTATTTTTAGAGAACATCCACTTTGTTATATTTGGgagct	630Δ*erm* 3'-CCA-sp6CRISPR3/16-BamHI
AM25	cCCATATCCTTGTATTTGCCTAACGCGATATAGAGCAAAAAg	630Δ*erm* 5'-CCA-

		sp3CRISPR12-SacI
AM26	gatccTTTTTGCTCTATATCGCGTTAGGCAAATACAAGGATATGGgagct	630Δ*erm* 3'-CCA-sp3CRISPR12-BamHI
AM27	cCCACGTATTAACACCTGTTCGGCACTATCACAATAAACACAAg	630Δ*erm* 5'-CCA-sp6CRISPR12-SacI
AM28	gatccTTGTGTTTATTGTGATAGTGCCGAACAGGTGTTAATACGTGGgagct	630Δ*erm* 3'-CCA-sp6CRISPR12-BamHI
OS707	cCCAGTTGTAAGAAGTATCATTCTATTTTTTAATCTTTCTg	R20291 5'-CCA-sp1CRISPR13-SacI
OS708	gatccAGAAAGATTAAAAAATAGAATGATACTTCTTACAACTGGgagct	R20291 3'-CCA-sp1CRISPR13-BamHI
OS709	cCCTGTTGTAAGAAGTATCATTCTATTTTTTAATCTTTCTg	R20291 5'-CCT-sp1CRISPR13-SacI
OS710	gatccAGAAAGATTAAAAAATAGAATGATACTTCTTACAACAGGgagct	R20291 3'-CCT-sp1CRISPR13-BamHI
OS711	cCCCGTTGTAAGAAGTATCATTCTATTTTTTAATCTTTCTg	R20291 5'-CCC-sp1CRISPR13-SacI
OS712	gatccAGAAAGATTAAAAAATAGAATGATACTTCTTACAACGGGgagct	R20291 3'-CCC-sp1CRISPR13-BamHI
OS713	cCCGGTTGTAAGAAGTATCATTCTATTTTTTAATCTTTCTg	R20291 5'-CCG-sp1CRISPR13-SacI
OS714	gatccAGAAAGATTAAAAAATAGAATGATACTTCTTACAACCGGgagct	R20291 3'-CCG-sp1CRISPR13-BamHI
OS715	cGAGGTTGTAAGAAGTATCATTCTATTTTTTAATCTTTCTg	R20291 5'-GAG-sp1CRISPR13-SacI
OS716	gatccAGAAAGATTAAAAAATAGAATGATACTTCTTACAACCTCgagct	R20291 3'-GAG-sp1CRISPR13-BamHI
OS717	cAATGTTGTAAGAAGTATCATTCTATTTTTTAATCTTTCTg	R20291 5'-AAT-sp1CRISPR13-SacI
OS718	gatccAGAAAGATTAAAAAATAGAATGATACTTCTTACAACATTgagct	R20291 3'-AAT-sp1CRISPR13-BamHI
OS719	cCCA**a**TTGTAAGAAGTATCATTCTATTTTTTAATCTTTCTg	R20291 5'-CCA-mut-

		sp1CRISPR13-SacI
OS720	gatccAGAAAGATTAAAAAATAGAATGATACTTCTTACAA**t**TGGgagct	R20291 3'-CCA-mut-sp1CRISPR13-BamHI
AM263	cCCCGTAGAGTCTTTATATGGTAGAGGTGGAATATATAAGTg	630Δ*erm* 5'-CCC-sp1CRISPR3/16-SacI
AM264	gatccACTTATATATTCCACCTCTACCATATAAAGACTCTACGGGgagct	630Δ*erm* 3'-CCC-sp1CRISPR3/16-BamHI
AM265	cCCGGTAGAGTCTTTATATGGTAGAGGTGGAATATATAAGTg	630Δ*erm* 5'-CCG-sp1CRISPR3/16-SacI
AM266	gatccACTTATATATTCCACCTCTACCATATAAAGACTCTACCGGgagct	630Δ*erm* 3'-CCG-sp1CRISPR3/16-BamHI
AM267	cTCAGTAGAGTCTTTATATGGTAGAGGTGGAATATATAAGTg	630Δ*erm* 5'-TCA-sp1CRISPR3/16-SacI
AM268	gatccACTTATATATTCCACCTCTACCATATAAAGACTCTACTGAgagct	630Δ*erm* 3'-TCA-sp1CRISPR3/16-BamHI
AM269	cTCTGTAGAGTCTTTATATGGTAGAGGTGGAATATATAAGTg	630Δ*erm* 5'-TCT-sp1CRISPR3/16-SacI
AM270	gatccACTTATATATTCCACCTCTACCATATAAAGACTCTACAGAgagct	630Δ*erm* 3'-TCT-sp1CRISPR3/16-BamHI
AM271	cTCGGTAGAGTCTTTATATGGTAGAGGTGGAATATATAAGTg	630Δ*erm* 5'-TCG-sp1CRISPR3/16-SacI
AM272	gatccACTTATATATTCCACCTCTACCATATAAAGACTCTACCGAgagct	630Δ*erm* 3'-TCG-sp1CRISPR3/16-BamHI
AM273	cTCCGTAGAGTCTTTATATGGTAGAGGTGGAATATATAAGTg	630Δ*erm* 5'-TCC-sp1CRISPR3/16-SacI
AM274	gatccACTTATATATTCCACCTCTACCATATAAAGACTCTACGGAgagct	630Δ*erm* 3'-TCC-sp1CRISPR3/16-BamHI
AM275	cTCAGTTGTAAGAAGTATCATTCTATTTTTTAATCTTTCTg	R20291 5'-TCA-sp1CRISPR13-SacI1
AM276	gatccAGAAAGATTAAAAAATAGAATGATACTTCTTACAACTGAgagct	R20291 3'-TCA-sp1CRISPR13-BamHI
AM277	cTCTGTTGTAAGAAGTATCATTCTATTTTTTAATCTTTCTg	R20291 5'-TCT-

		sp1CRISPR13-SacI
AM278	gatccAGAAAGATTAAAAAATAGAATGATACTTCTTACAACAGAgagct	R20291 3'-TCT-sp1CRISPR13-BamHI
AM279	cTCGGTTGTAAGAAGTATCATTCTATTTTTTAATCTTTCTg	R20291 5'-TCG-sp1CRISPR13-SacI
AM280	gatccAGAAAGATTAAAAAATAGAATGATACTTCTTACAACCGAgagct	R20291 3'-TCG-sp1CRISPR13-BamHI1
AM281	cTCCGTTGTAAGAAGTATCATTCTATTTTTTAATCTTTCTg	R20291 5'-TCC-sp1CRISPR13-SacI
AM282	gatccAGAAAGATTAAAAAATAGAATGATACTTCTTACAACGGAgagct	R20291 3'-TCC-sp1CRISPR13-BamHI
cas* genes cloning into pRPF185Δ*gus		
AM183	TTACATgagctcTTTTGTGAAGGAGGAGTAACAATG	CD2975-2976 (cas2-1) SacI F
AM184	TTACATgagctcAACAATGTGGCTAAAATTTAC	CD2975-2977 (cas2-1-4) SacI F
AM185	GCACATggatccACCTATATAAACATTTCATCAGAATAC	CD2975-76 (cas2-1) and CD2975-2977 (cas2-1-4) BamHI R
**CRISPR adaptation detection and analysis**		
PB142	GGAAATACTAAGTTTATTTTGGG	CRISPR3-4/16-15 F
PB143	ACTTATATATTCCACCTCTACCA	CRISPR3-4/16-15 R
PB144	GGAAATACTCAATTTATTTTGGG	CRISPR6 F
PB145	AGTGATGAATGTTCAGAAGAGGA	CRISPR6 R
PB146	GAAAATGCCCAGTTTATTTTGGG	CRISPR7 F
PB147	AATAATTGCAGTATCTTTTAAGA	CRISPR7 R
PB148	GAAAATACTAAGTTTATTTTGGG	CRISPR8 F
PB149	GAATTATTAGAATCATTTAATAT	CRISPR8 R
PB150	GAAAATACTTAGTTTATTTTGGG	CRISPR9 F
PB151	CTTGCAAGAATGGTTTTAATAAT	CRISPR9 R
PB152	GGAGATGCTAAGTTTATTTTGGA	CRISPR10 F
PB153	TTAAGACTAGCAGACTCATAAGC	CRISPR10 R
PB154	GGAAATGCTAAGTTTATTTTGGG	CRISPR11 F
PB155	AAAATAAGCTATGAAGCTAAAGG	CRISPR11 R
PB156	GGAAATACTCAATTTATTTTGGG	CRISPR12 F
PB157	TATTCCCGAAACTGAAAGAAATC	CRISPR12 R
PB158	TATTCAAATATACCTATTTTGGG	CRISPR17 F
PB159	AAATTGAAGTAATAAAGAAAATA	CRISPR17 R
AM228	tcgtcggcagcgtcagatgtgtataagagacagAATACTAAGTTTATTTTGG	CRISPR8 nested

	GGTT	PCR Illumina F
AM234	gtctcgtgggctcggagatgtgtataagagacagAGAATCATTTAATATACCAATAATAG	CRISPR8 nested PCR Illumina R
AM235	tcgtcggcagcgtcagatgtgtataagagacagAATACTTAGTTTATTTTGGGGTT	CRISPR9 nested PCR Illumina F
AM236	gtctcgtgggctcggagatgtgtataagagacagTGGTTTTAATAATGAGTTCTAATTC	CRISPR9 nested PCR Illumina R
**qRT-PCR**		
QRTBD37	GGGAGACTTGAGTGCAGGAG	16S RNA qPCR F
QRTBD38	GTGCCTCAGCGTCAGTTACA	16S RNA qPCR R
AM289	GAGAGAATTGTATAGATGTAAGTGTTG	CRISPR6 qPCR F
OS679	GCAGTGAGCAATATTTGCGATA	CRISPR3-4/16-15 qPCR F
OS680	CAAATTTGCAGTGAACCATGA	CRISPR3-4/16-15 qPCR R
AM290	GTGATGAATGTTCAGAAGAGGA	CRISPR6 qPCR R
AM291	AAGCTTTATCATTTGCACTACTC	CRISPR7 qPCR F
AM292	CAGTATCTTTTAAGAATTGAGTGGTT	CRISPR7 qPCR R
AM175	TGCAAATTTAAGAGAGTTGTATACG	CRISPR8 qPCR F
AM176	TATCTTGAGCTGTCAATGTGAAC	CRISPR8 qPCR R
AM293	GGATTGAGGGTGTGTGATAAA	CRISPR9 qPCR F
AM294	CTTGCAAGAATGGTTTTAATAATGAG	CRISPR9 qPCR R
PB152	GGAGATGCTAAGTTTATTTTGGA	CRISPR10 F
PB153	TTAAGACTAGCAGACTCATAAGC	CRISPR10 R
OS472	CCATTGATTTCTTTCAGTTTCG	CRISPR12 qPCR F
OS473	CGCGTTAGGCAAATACAAGG	CRISPR12 qPCR R
AM177	TCGCTCACTGCAAATTTTG	CRISPR17 qPCR F
AM178	AAACGCAGGTCAAACCTTA	CRISPR17 qPCR R
