# Supplementary material for: Protospacer-Adjacent Motif Specificity during Clostridioides difficile Type I-B CRISPR-Cas Interference and Adaptation
Source: mBio. 2021 Aug 24;12(4):e02136-21. doi: 10.1128/mBio.02136-21 (PMC8406132; doi:10.1128/mBio.02136-21)
Supplement: TABLE S3 [file mbio.02136-21-st003.pdf]

Table S3A. Sequencing read counts for 630Δerm PAM libraries before and after conjugation.

PAM library	Number of raw reads	Number of reads containing PAM sequences
Before_the_conjugation	1805894	1374743
After_the_conjugation	1631633	1241220

Before_the_conjugation

PAM	Number of reads
TGT	28230
CAC	38798
GTA	12648
GAC	26446
GAT	25298
GGG	23980
AAG	32517
CAT	32142
CGG	28108
ATA	12305
GAA	14679
TAG	32877
TCA	14624
TAC	35925
TCG	25460
GGT	23043
TGG	29132
TCC	21678
CGT	23925
AGT	23143
ACC	17311
CAG	34314
CTC	23766
TGA	14480
AAT	26520
TGC	23191
CCA	11732
TTC	23416
CAA	18915
GGA	12071
ATG	22432
AAA	17661
TCT	18851
ATC	20560
GAG	29750
GTT	18697
TTG	23280
ACA	13265
ACG	19612
TAA	21065
CTG	21799
AGA	13080
GCT	14457

TTA	12886
AAC	28560
GTG	21183
CCG	20081
GCC	16939
AGC	18797
AGG	27701
ATT	17218
GTC	20480
CGC	23509
ACT	17876
GCG	17916
TTT	18549
CCT	17869
GCA	10379
TAT	29000
CTA	13826
GGC	21304
CTT	20152
CGA	14873
CCC	20462

After_the_conjugation

PAM	Number of reads
ATC	28788
CAC	64547
AGA	16402
TAC	44588
TTC	16903
GTA	78891
CAT	27933
TAT	30440
AAG	24892
TGA	12630
AAA	18073
GTG	17079
TAA	18958
GAG	26712
CTC	18343
ACT	13538
TCC	4171
CTG	15371
TTT	11800
TTG	16337
CAA	27034
ATT	30052
AGC	15264
ACC	8462
GTT	11993
CAG	34174

ATA	21119
GCT	14223
AGG	22760
AAC	24643
GGG	17996
AGT	15179
GGT	22448
CGC	32262
GCC	12913
CTA	15907
GAT	16977
TGC	19777
GCA	6842
ACG	16177
CTT	14426
GAA	35318
TGT	25192
TAG	43902
TGG	30767
GAC	30725
CGG	8500
ATG	24006
CGT	31333
GCG	9249
GGC	17336
AAT	24913
CCT	2114
GTC	12678
GGA	9798
ACA	7158
TTA	11106
CGA	4120
TCG	1459
CCG	1030
TCT	1068
CCA	620
CCC	1026
TCA	778

Table S3B. Sequencing read counts for R20291 PAM libraries before and after conjugation.

PAM library	Number of raw reads	Number of reads containing PAM sequences
Before_the_conjugation	2063043	1586293
After_the_conjugation	1592954	1263124

Before_the_conjugation

PAM	Number of reads
GGC	30304
TCC	7327
GTC	19008
TTA	27082
GAG	41392
GAC	23402
GCA	46322
AAA	37514
TGT	23569
GTT	27125
AGA	53014
GCG	28691
AGT	29010
GGA	97527
ATC	12279
GTA	58536
TGG	36911
ATG	23272
GGT	49007
GAA	59483
AAG	27136
CAA	22078
GTG	41602
ATA	31770
GGG	72131
TGC	15160
TTC	8018
TGA	48529
TAA	33123
CCG	10036
CGC	8390
ACG	20553
GCC	14987
TCG	16517
AGG	41256
CTT	7194
CTG	11679
CAT	10855
AAT	20410
GAT	30063
CGA	27221
AGC	19396
TAG	24494

TCA	22721
TAC	10835
ACT	11713
AAC	15526
ATT	14534
TAT	17475
TTG	18498
GCT	18067
CTA	16612
CTC	6108
CGG	19901
ACA	30363
TTT	13672
CGT	14189
CCT	6001
CCA	12801
CCC	4374
CAG	14248
TCT	9910
ACC	8598
CAC	6774

After_the_conjugation

PAM	Number of reads
AAA	41368
TGA	27100
TGT	3554
GCA	70262
ATT	9834
GAC	25953
TTA	15767
GGG	64305
GGA	52265
CGA	41460
GAA	35324
GCG	40487
ACG	13820
GAT	20503
GCT	23689
CAG	19553
AGC	32936
CTA	15276
GTC	13230
GGC	25256
TTG	10858
TGG	53746
CGG	18095
TAA	21333
TAT	14963
AGT	11960

AGA	32417
AGG	24582
GTT	15944
GTG	34706
GTA	78083
GAG	36015
GCC	22129
CAA	10232
AAG	27489
CTG	36258
GGT	32967
CAT	5789
AAT	14855
ACA	16896
AAC	9892
TAC	8506
ATA	26262
TTT	10021
ATG	9467
CTT	8204
TAG	8845
TGC	14077
ACC	12763
CTC	4403
CGC	5751
CGT	7791
ATC	9371
ACT	6406
TTC	4063
CAC	2628
CCA	447
TCC	2275
TCT	118
TCA	229
TCG	161
CCC	43
CCT	49
CCG	93
